# Inhibition of β-catenin signaling respecifies anterior-like endothelium into beating human cardiomyocytes

**DOI:** 10.1242/dev.117010

**Published:** 2015-09-15

**Authors:** Nathan J. Palpant, Lil Pabon, Meredith Roberts, Brandon Hadland, Daniel Jones, Christina Jones, Randall T. Moon, Walter L. Ruzzo, Irwin Bernstein, Ying Zheng, Charles E. Murry

**Affiliations:** 1 Department of Pathology, University of Washington School of Medicine, Seattle, WA 98109, USA; 2 Center for Cardiovascular Biology, University of Washington School of Medicine, Seattle, WA 98109, USA; 3 Institute for Stem Cell and Regenerative Medicine, University of Washington School of Medicine, Seattle, WA 98109, USA; 4 Department of Bioengineering, University of Washington School of Medicine, Seattle, WA 98109, USA; 5 Department of Pediatrics, University of Washington School of Medicine, Seattle, WA 98109, USA; 6 Clinical Research Division, Fred Hutchinson Cancer Research Center, Seattle, WA 98109, USA; 7 Department of Computer Science and Engineering, University of Washington School of Medicine, Seattle, WA 98109, USA; 8 Department of Pharmacology, University of Washington School of Medicine, Seattle, WA 98109, USA; 9 Howard Hughes Medical Institute, Seattle, WA 98109, USA; 10 Department of Medicine/Cardiology, University of Washington School of Medicine, Seattle, WA 98109, USA

**Keywords:** Cardiac, Hematopoiesis, Endothelium, Human embryonic stem cell, Differentiation

## Abstract

During vertebrate development, mesodermal fate choices are regulated by interactions between morphogens such as activin/nodal, BMPs and Wnt/β-catenin that define anterior-posterior patterning and specify downstream derivatives including cardiomyocyte, endothelial and hematopoietic cells. We used human embryonic stem cells to explore how these pathways control mesodermal fate choices *in vitro.* Varying doses of activin A and BMP4 to mimic cytokine gradient polarization in the anterior-posterior axis of the embryo led to differential activity of Wnt/β-catenin signaling and specified distinct anterior-like (high activin/low BMP) and posterior-like (low activin/high BMP) mesodermal populations. Cardiogenic mesoderm was generated under conditions specifying anterior-like mesoderm, whereas blood-forming endothelium was generated from posterior-like mesoderm, and vessel-forming CD31^+^ endothelial cells were generated from all mesoderm origins. Surprisingly, inhibition of β-catenin signaling led to the highly efficient respecification of anterior-like endothelium into beating cardiomyocytes. Cardiac respecification was not observed in posterior-derived endothelial cells. Thus, activin/BMP gradients specify distinct mesodermal subpopulations that generate cell derivatives with unique angiogenic, hemogenic and cardiogenic properties that should be useful for understanding embryogenesis and developing therapeutics.

## Abstract (precis)

**Summary:** The manipulation of signals that control embryonic patterning allows human pluripotent stem cells to be differentiated into endothelial subpopulations with distinct haematopoietic, angiogenic and cardiogenic potential.

## INTRODUCTION

The anterior-posterior axis is the earliest to form in the embryo and is evolutionarily most ancient (Kimelman and Martin, 2012). Anterior primitive streak gives rise to endoderm, whereas the mid- to posterior primitive streak gives rise to different mesodermal lineages. Anterior mesoderm (mid-streak) gives rise to cardiac and endocardial endothelium, whereas posterior mesoderm (posterior streak) gives rise to the blood-forming endothelium and vasculature (Murry and Keller, 2008).

Well-described anterior-posterior morphogen gradients, including those of activin A/nodal and BMP4, are thought to pattern mesoderm subtypes (Nostro et al., 2008; Sumi et al., 2008; Kattman et al., 2011). Such gradients are proposed to specify anterior mesodermal fates like cardiomyocytes versus posterior mesodermal fates like blood. Remarkably, a recent study showed that ectopic induction of a nodal/BMP gradient in zebrafish embryos was sufficient to create an entirely new embryonic axis that could run parallel, anti-parallel or orthogonal to the primary axis (Xu et al., 2014). This provides compelling evidence that nodal and BMP are significant determinants of anterior-posterior patterning in the embryo.

Functionally, the TGFβ signaling family members activin A and BMP4 synergistically activate Wnt/β-catenin signaling during primitive streak (PS) formation, and are essential for establishing downstream lineages (Nostro et al., 2008; Sumi et al., 2008). Stimulation of the Wnt/β-catenin pathway during gastrulation is required to form mesoderm (Yamaguchi et al., 1999; Lekven et al., 2001), and the subsequent Wnt/β-catenin signaling gradients are thought to be responsible for specifying sublineages of mesodermal derivatives (Martin and Kimelman, 2012). Recent work has shown that Wnt/β-catenin signaling is involved in redirecting fate choices between mesodermal lineages in the post-gastrulation stage of development (Schoenebeck et al., 2007; Van Handel et al., 2012; Palpant et al., 2013).

In the present study we investigated whether unique functional derivatives of mesoderm could be generated from human pluripotent stem cells (hPSCs) *in vitro* by processes that reflect embryological patterning during gastrulation. We modulated activin A, BMP4 and Wnt/β-catenin signaling in order to manipulate key cell fate transitions from the undifferentiated state to mature cell types. Cardiomyocytes were derived efficiently from anterior-like mesoderm, and blood more efficiently from posterior-like mesoderm. Endothelium was generated from all mesodermal subtypes studied. These endothelial subpopulations exhibit differences in hematopoietic, angiogenic, and cardiogenic potential, reflecting influences of their developmental ontogeny.

## RESULTS

### Patterning mesoderm *in vitro* using activin A/BMP4

**Fig. 1.**
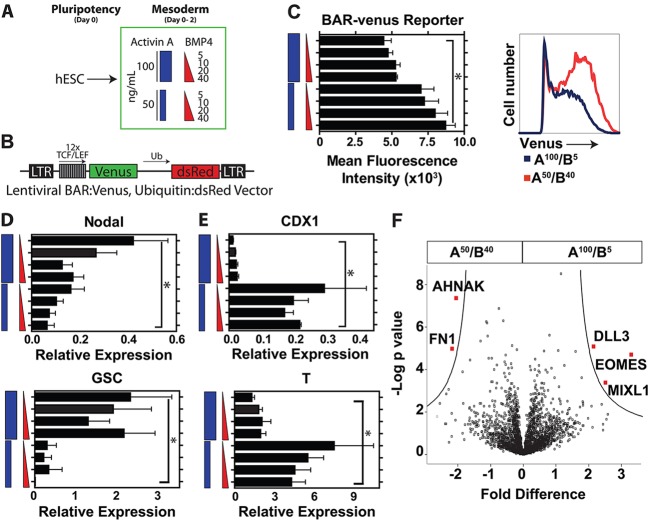
**Directing mesoderm patterning by titrating activin A and BMP4.** (A) The experimental approach for directing undifferentiated hESCs into anterior versus posterior mesoderm using doses of activin A and BMP4. (B) The BAR-Venus:Ub-dsRed vector used to measure endogenous Wnt/β-catenin signaling in differentiating hESCs. (C) Changes in mean fluorescence intensity of BAR-Venus activity on day 2 of directed differentiation under different activin A/BMP4 conditions (left), and a representative flow plot showing reporter activity in conditions of 100 ng/ml activin A and 5 ng/ml BMP4 (A^100^/B^5^) versus 50 ng/ml activin A and 40 ng/ml BMP4 (A^50^/B^40^) (right). (D,E) qRT-PCR analysis of genes involved in mesoderm patterning, including anterior mesoderm markers *NODAL* and *GSC* (D) as well as posterior markers *CDX1* and brachyury (*T*) (E). *n*=5 biological replicates for BAR-Venus activity and qPCR analysis. *P<0.05. (F) Volcano plot showing proteins significantly differentially expressed between day 2 A^50^/B^40^ and A^100^/B^5^ cells using an FDR threshold of 0.05. For mass spectrometry *n*=2 biological replicates, each with three technical replicates per sample.

Inspired by the dominant role of activin A and BMP4 in establishing the anterior-posterior axis of the embryo (Sumi et al., 2008; Xu et al., 2014), we hypothesized that titrating activin A and BMP4 would modulate the strength of Wnt/β-catenin signaling and thereby polarize mesoderm specification from undifferentiated human embryonic stem cells (hESCs) along the anterior-posterior axis (Fig. 1A). To analyze Wnt/β-catenin signaling activity in mesoderm patterning, we used a RUES2 hESC line that expresses the green fluoroprotein Venus under control of multimerized TCF/LEF elements (β-catenin-activated reporter; BAR-Venus:UB-dsRed), as previously described (Davidson et al., 2012; Palpant et al., 2013). We chose to monitor the activity of the pathway through the BAR-Venus reporter in combination with gene expression of Wnt modulatory proteins during directed differentiation.

We tested the hypothesis that modulating activin A and BMP4 signaling could induce different types of mesoderm. hESCs were differentiated using activin A at either 100 ng/ml (A^100^) or 50 ng/ml (A^50^) in combination with a dose range of BMP4 between 5 ng/ml and 40 ng/ml (B^5^-B^40^) (a detailed schematic outlining the differentiation methods employed in this manuscript is shown in supplementary material Fig. S1). We found that concentrations lower than 50 ng/ml activin A did not markedly influence mesoderm specification (supplementary material Fig. S2). Based on previous work on directed differentiation from hESCs, we have established that mesoderm is specified on day 2 of differentiation (Paige et al., 2010, 2012; Palpant et al., 2013). At this time point, analysis of Venus activity showed that differentiation with A^100^ caused significantly lower activity of endogenous Wnt/β-catenin signaling than A^50^ (Fig. 1A-C). Consistent with this, analysis of Wnt/β-catenin signaling modulators showed high mRNA expression of *WNT3A* and *WNT8A* in conditions of A^50^, with increased levels of the Wnt/β-catenin signaling inhibitor *DKK1* predominantly in conditions of A^100^ (supplementary material Fig. S3A). By contrast, increasing BMP4 concentrations only modestly increased Wnt/β-catenin reporter activity and did not significantly change the expression of Wnt regulators (Fig. 1C; supplementary material Fig. S3A).

Other modulators of mesoderm patterning were analyzed by qRT-PCR, which showed that the pan-mesoderm markers *KDR* (*VEGFR2*) and *MESP1* are expressed across all conditions (supplementary material Fig. S4A). Genes involved in anterior mesendoderm development, including those encoding the bicoid homeobox protein goosecoid (GSC) and NODAL, were more highly expressed in conditions of A^100^ (Fig. 1D). This is consistent with studies showing that NODAL functionally interacts with Wnt factors to activate genes, such as *GSC*, that are required for anterior PS patterning and germ layer formation (Reid et al., 2012). Conversely, genes more highly expressed in posterior mesoderm, including brachyury (*T*) and *CDX1*, a key modulator of Hox gene activity (Lengerke et al., 2011) necessary for blood development (Wang et al., 2008), were expressed more highly in conditions of A^50^ (Fig. 1E).

Using protein mass spectrometry, we analyzed day 2 mesoderm generated by different induction approaches. These data show a high degree of similarity between the mesodermal subtypes, with the exception of several molecules that are significantly different and reinforce the observation that proteins involved in anterior versus posterior mesoderm patterning are appropriately expressed in a lineage-specific manner (Fig. 1F). Specifically, fibronectin 1 (FN1) was highly expressed under conditions of A^50^/B^40^. Although FN1 is functionally required for organ development in a wide range of tissues, it is functionally required in posterior but not anterior lateral plate mesoderm development during gastrulation (George et al., 1993). Furthermore, the T-box transcription factor eomesodermin (EOMES) acts upstream of MESP1 to direct cardiogenic mesoderm (Costello et al., 2011) and MIXL1 is functionally required for the morphogenesis of axial mesoderm and represses posterior mesoderm fates (Hart et al., 2002). As such, these proteins are appropriately expressed in conditions of A^100^/B^5^.

Taken together, gene expression analysis, proteomic analysis, and Wnt/β-catenin signaling activity support the notion that modulating the concentration of activin A/BMP4 can polarize mesoderm along the anterior-posterior axis *in vitro* from human pluripotent stem cells.

### Specification of cardiogenic mesoderm from anterior mesoderm

Using this dosing regimen of activin A/BMP4, we next sought to directly assess the effect on downstream mesodermal derivatives using cardiomyocytes as readouts of anterior differentiation. The protocol for cardiac directed differentiation is based on studies from our laboratory and others showing that cardiac specification involves a biphasic modulation of Wnt/β-catenin signaling. Specifically, robust Wnt/β-catenin signaling activation is required to direct mesoderm, and specification into the cardiac lineage involves downregulation of Wnt/β-catenin signaling (Ueno et al., 2007; Paige et al., 2010; Lian et al., 2012; Palpant et al., 2013). The protocol used for directing cardiac differentiation is detailed in the supplementary Materials and Methods and Fig. S1.

**Fig. 2.**
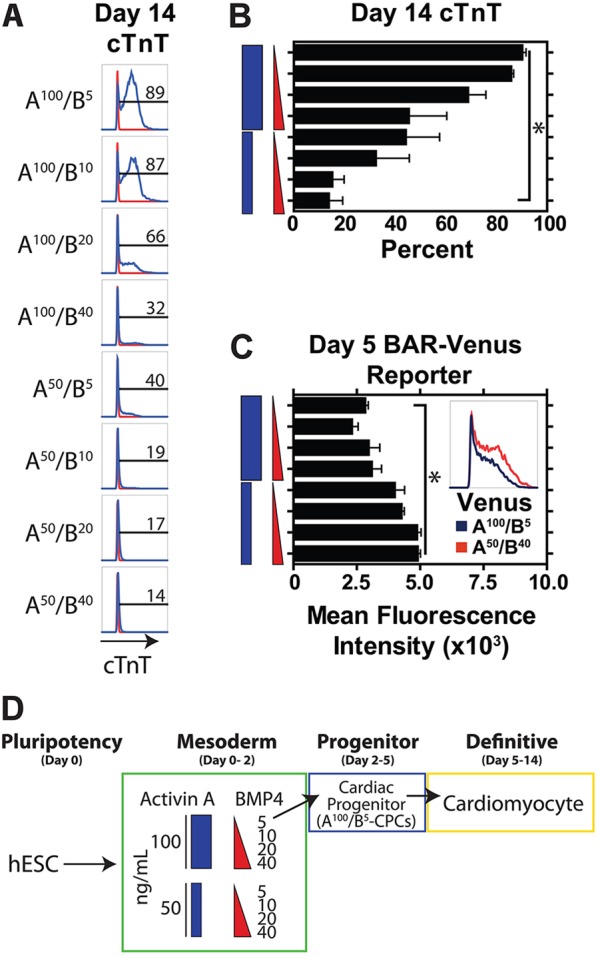
**High activin A and low BMP4 concentrations direct cardiac differentiation.** (A,B) Flow cytometry for cTnT on day 14 of differentiation across the range of differentiation conditions defined by doses of activin A and BMP4, with raw histograms (A) and mean data across five biological replicates (B) showing the highest efficiency of cardiac differentiation (percentage cTnT^+^ cardiomyocytes) in conditions of A^100^/B^5^. (C) Wnt/β-catenin signaling activity as measured by the BAR-Venus reporter in day 5 CPCs under different doses of activin A and BMP4. Inset shows raw flow cytometry plot of Venus activity in A^100^/B^5^ versus A^50^/B^40^ CPCs. (D) Schematic illustrating that cardiomyocytes are generated optimally from A^100^/B^5^ CPCs. *n*=5 biological replicates. *P<0.05.

Analysis at day 14 showed that the highest efficiency of cardiac differentiation occurred under conditions of A^100^/B^5^ [90±1% cTnT (TNNT2)^+^ cardiomyocytes] (Fig. 2A-D). By contrast, cardiomyocyte differentiation progressively decreased with lower doses of activin A and higher doses of BMP4, with purity dropping as low as 14±6% cTnT^+^ cells when initiated under conditions of A^50^/B^40^ (Fig. 2A,B). Analysis of day 5 cardiac progenitor cells (CPCs) did not show any correlation of KDR^+^/PDGFRα^+^ cells and cardiac differentiation efficiency under these differentiation conditions (supplementary material Fig. S4B,C).

To determine the effect of induction on cardiac cell fate specification, we compared the expression profiles of day 14 cells induced with either A^100^/B^5^ or A^50^/B^40^, with subsequent differentiation under cardiogenic conditions (supplementary material Fig. S5). RNA-seq data indicate that A^100^/B^5^ cells give rise to a cardiac phenotype as expected. By contrast, A^50^/B^40^ cells at day 14 showed a robust endothelial phenotype. These data indicate that induction conditions are deterministic with regard to fate potential, such that cardiogenic mesoderm is specified early from A^100^/B^5^ conditions and an endothelial fate is specified from A^50^/B^40^ conditions.

To understand the role of Wnt/β-catenin signaling under cardiac differentiation conditions, we analyzed BAR-Venus reporter activity in day 5 CPCs (Fig. 2C; supplementary material Fig. S3B,D). Compared with the peak of mesoderm formation at day 2, there was a significant downregulation of Wnt/β-catenin signaling in day 5 cells, with the greatest downregulation occurring in CPCs initiated under A^100^ conditions (Fig. 2C; supplementary material Fig. S3D). Compared with day 2, gene expression analysis of Wnt modulators in day 5 CPCs showed a significant downregulation of canonical Wnt ligands but a marked increase in canonical Wnt inhibitors including *TMEM88* and *WNT5A* in A^100^ conditions (supplementary material Fig. S3B). Importantly, we also observed a significant increase in expression of the canonical Wnt/β-catenin inhibitor *DKK1* in anterior-derived cardiogenic mesoderm, which suggests a more repressive state of Wnt/β-catenin signaling during cardiac specification (supplementary material Fig. S3B).

### Derivation of endothelium

Given that factors involved in hemato-endothelial mesoderm development, such as CDX1 and WNT8A, had an inverse correlation with cardiac differentiation efficiency (Fig. 1E; supplementary material Fig. S3A), we hypothesized that this mesodermal differentiation platform could be used to direct downstream vascular fate choices and efficiently generate blood and endothelial cells. Since endothelium is generated from all mesodermal origins (Murry and Keller, 2008), we sought to understand the impact on cell fate decisions of endothelial development after initiating differentiation with different concentrations of activin A and BMP4. We tested a wide range of culture conditions involving stimulation with major determinants of endothelial fate, including varying the timing and concentrations of VEGF, BMP4, CHIR-99021 and FGF, based on previous studies describing endothelial differentiation from PSCs (Kennedy et al., 2007, 2012; Choi et al., 2012; Rafii et al., 2013; White et al., 2013) (supplementary material Fig. S7; data not shown). Studies from our lab and others have established a role for Wnt/β-catenin signaling in specifying endothelial commitment (Woll et al., 2008; Palpant et al., 2013; Sturgeon et al., 2014). We observed, however, that addition of the small molecule Wnt/β-catenin agonist CHIR-99021 between days 2 and 5 inhibited endothelial fate specification, indicating that other factors involved in the specification of endothelium tightly orchestrate the dosage of Wnt/β-catenin signaling (supplementary material Fig. S7). We describe in detail our protocol for endothelial differentiation in the supplementary Materials and Methods and Fig. S1.

**Fig. 3.**
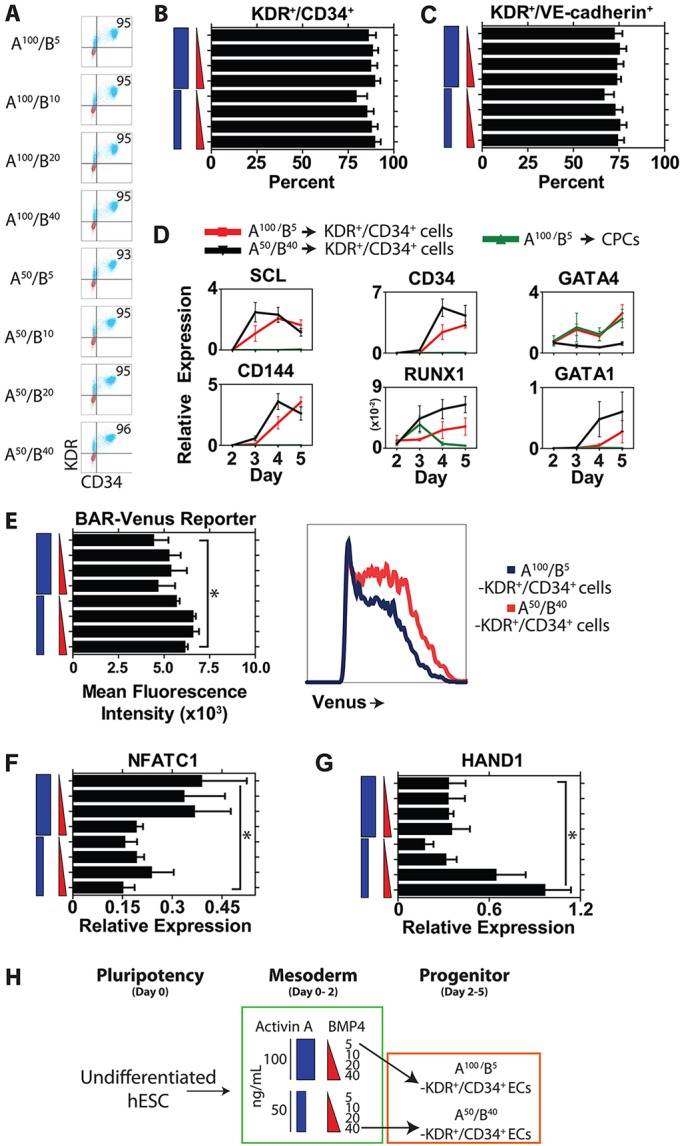
**KDR^+^/CD34^+^/VE-cadherin^+^ cells are generated efficiently across doses of activin A and BMP4.** (A-C) Flow cytometry plots (A) and mean data for the percentage of cells double positive for KDR and CD34 (B) or for KDR and VE-cadherin (C). (D) qRT-PCR analysis across the timecourse of days 2-5 of differentiation comparing A^100^/B^5^-derived CPCs with A^100^/B^5^-derived KDR^+^/CD34^+^ cells and A^50^/B^40^-derived KDR^+^/CD34^+^ cells. CD144 refers to VE-cadherin. (E) Wnt/β-catenin signaling activity as measured by the BAR-Venus reporter in day 5 KDR^+^/CD34^+^ cells under different doses of activin A and BMP4 (left), with representative raw flow cytometry plot of Venus activity in A^100^/B^5^ KDR^+^/CD34^+^ versus A^50^/B^40^ KDR^+^/CD34^+^ cells (right). (F,G) qRT-PCR analysis for markers of endothelium including *NFATC1* (F) and *HAND1* (G). (H) Schematic showing lineage differentiation for directing undifferentiated hESCs into A^100^/B^5^-derived KDR^+^/CD34^+^ cells and A^50^/B^40^-derived KDR^+^/CD34^+^ cells. *n*=3-8 replicates per sample for all assays. *P<0.05.

In our optimized protocol, analysis of pan-endothelial markers on day 5 of differentiation showed that we could generate greater than 90% KDR^+^/CD34^+^ double-positive cells (Fig. 3A,B). These data show that a high percentage of KDR^+^/CD34^+^ cells can be generated across the full spectrum of activin A and BMP4 conditions used to establish mesoderm patterning at the onset of differentiation. Consistent with endothelial development, a high percentage of these cells express VE-cadherin (Fig. 3C). We next assessed whether these endothelial cells exhibited molecular characteristics of developing endothelium (Shalaby et al., 1997; Wu et al., 2011; Nakano et al., 2013). Key markers involved in vascular and blood development, including the master transcription factor of hemato-endothelial development SCL (TAL1), as well as VE-cadherin (CD144, or CDH5), CD34, RUNX1 and GATA1, were significantly upregulated between days 2 and 5 in both populations of KDR^+^/CD34^+^ cells, but not under cardiac differentiation conditions (Fig. 3D).

Analysis of BAR-Venus activity indicated that Wnt/β-catenin signaling was markedly elevated in day 5 KDR^+^/CD34^+^ cells compared with day 5 CPCs (supplementary material Fig. S3D). Furthermore, the posterior A^50^/B^40^ KDR^+^/CD34^+^ cells had significantly higher Wnt/β-catenin signaling activity than the anterior A^100^/B^5^ KDR^+^/CD34^+^ cells (Fig. 3E; supplementary material Fig. S3D). Analysis of Wnt regulatory molecules indicated that, although expression of the Wnt ligands WNT3A and WNT8A was reduced in KDR^+^/CD34^+^ cells, as seen in cardiac differentiation, the expression levels of Wnt/β-catenin signaling inhibitors such as TMEM88, WNT5A and DKK1 were reduced in KDR^+^/CD34^+^ cells compared with CPCs (supplementary material Fig. S3A-C). These data indicate that Wnt/β-catenin signaling dosage does not appear to influence endothelial differentiation, but we hypothesized that differences in Wnt/β-catenin signaling might be crucial for specifying endothelial subtypes.

In keeping with the hypothesis that Wnt/β-catenin signaling dosage may participate in the specification of different endothelial subtypes, we analyzed molecular markers of endocardial versus vascular endothelial fate. Recent fate-mapping data have suggested that NFATC1 and GATA4 are markers of anterior-derived endocardial endothelium and distinguish it from posterior-derived vascular hemogenic endothelium (Misfeldt et al., 2009; Peterkin et al., 2009). Consistent with this, *Nfatc1* knockout mice die mid-gestation due to endocardial valve malformation-induced heart failure (de la Pompa et al., 1998; Ranger et al., 1998; Misfeldt et al., 2009; Wu et al., 2011). We found the cardiogenic factor GATA4 expressed only in anterior-derived mesodermal derivatives (Fig. 3D) and NFATC1 was significantly elevated in anterior A^100^/B^5^ KDR^+^/CD34^+^ cells (Fig. 3F).

By contrast, lineage-tracing studies have identified a role for HAND1 in cardiomyocyte development and posterior mesoderm development but not in endocardial development (Morikawa and Cserjesi, 2004; Barnes et al., 2010; Maska et al., 2010). Reflecting this cell fate, we found that HAND1 is significantly upregulated in the posterior A^50^/B^40^ KDR^+^/CD34^+^ cells (Fig. 3G). We analyzed the expression of additional vascular development markers known to be involved in arterial specification, including SOX17, EFNB2 and CXCR4. However, we found no difference in the expression of these molecules based on induction method (supplementary material Fig. S4D).

Taken together, these data show that KDR^+^/CD34^+^ cells can be generated with equal efficiency across a range of activin A/BMP4 dosing regimens with molecular differences that suggest an endocardial versus vascular endothelial lineage fate (Fig. 3H).

### Anterior and posterior endothelial cells exhibit overlapping as well as lineage-specific endothelial function

**Fig. 4.**
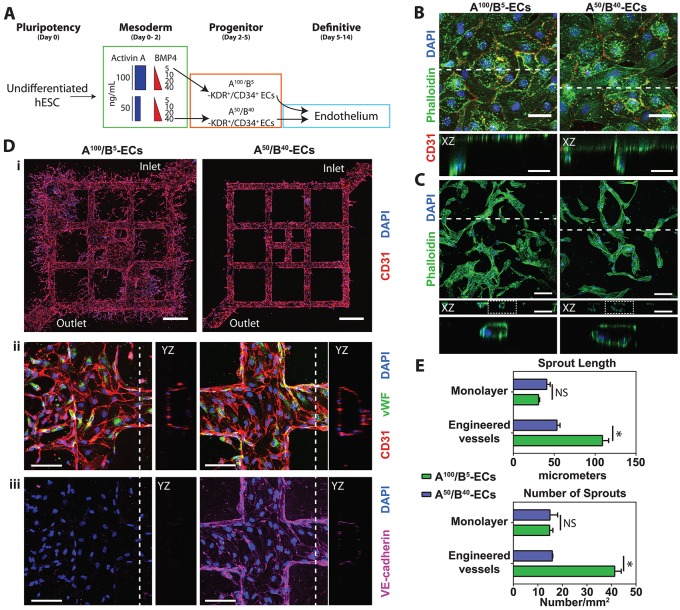
**KDR^+^/CD34^+^ cells can differentiate into functional endothelium.** (A) Experimental approach for directing undifferentiated hESCs into A^100^/B^5^ and A^50^/B^40^ ECs and then into differentiated and functional ECs. (B,C) Day 14 A^100^/B^5^ and A^50^/B^40^ ECs were plated onto collagen as a monolayer to assay sprouting angiogenic potential (B) or embedded into collagen to assay tube formation potential (C). Orthogonal views (at the dashed line) are shown beneath as a *z*-stack in the *xz* axis. (D) Day 14 A^100^/B^5^ and A^50^/B^40^ ECs were seeded into engineered microvessels (i) and exposed to flow for 4 days. Engineered microvessels were assayed for vessel structure and cellular expression of VWF and CD31 (ii) as well as VE-cadherin (iii) together with a nuclear counterstain. Orthogonal views are shown to the side as a *z*-stack of the *yz* axis. (E) Quantification of the number and length of sprouting vessels in monolayer and engineered vessels. *n*≥3 replicates per sample. *P<0.05; NS, not significant. Scale bars: 100 µm, except 500 µm in Di.

On the basis of these findings, we carried out secondary assays to assess the functionality of day 5 A^100^/B^5^ and A^50^/B^40^ endothelial cells (ECs) (Fig. 4). CD34^+^ sorted or unsorted day 5 cells were plated on gelatin-coated plates in endothelial growth medium (EGM) containing VEGF, bFGF (FGF2) and the GSK3 inhibitor CHIR-99021 for 9 days. Under these conditions, A^100^/B^5^ and A^50^/B^40^ ECs both gave rise to greater than 95% pure CD31 (PECAM1)^+^ ECs by flow cytometry analysis (supplementary material Fig. S8A). Culturing day 5 cells after FACS for CD34^+^ cells did not improve CD31^+^ cell purity compared with unsorted cells (supplementary material Fig. S8A). Both populations of ECs showed expression of appropriate markers, including CD31 and the endothelial marker von Willebrand factor (VWF), and no significant difference in proliferation (supplementary material Fig. S8B,C).

We carried out functional assays to determine whether differentiated ECs from both lineages showed evidence of sprouting angiogenesis or tube formation *in vitro.* Day 14 A^100^/B^5^ and A^50^/B^40^ ECs were plated as a monolayer on collagen (2 mg/ml) to assess gel invasion/sprouting or embedded within collagen to assess tube formation (Fig. 4B,C). Orthogonal views show that both populations of ECs have the capacity for sprouting angiogenesis based on invasion into the collagen matrix and tube formation based on *de novo* lumen formation (Fig. 4B,C).

To assess the ability of these cells to respond to fluid shear stress, we analyzed the function of day 14 A^100^/B^5^ and A^50^/B^40^ ECs when seeded into microfabricated engineered vessels as previously described (Zheng et al., 2012) (Fig. 4D). The cells were seeded into microfluidic channels to form a templated microvascular network with a perfusable lumen and cultured for 4 days under gravity-driven flow. Surprisingly, A^100^/B^5^ ECs showed robust sprouting activity with *de novo* angiogenic vessels. By contrast, A^50^/B^40^ ECs had minimal angiogenic potential under the same conditions (Fig. 4Di). Immunohistochemical analysis of these engineered vessels showed similar expression of CD31 and VWF in both populations (Fig. 4Dii). By contrast, under these conditions, expression of VE-cadherin was found exclusively within the non-sprouting A^50^/B^40^ ECs (Fig. 4Diii), consistent with the finding that shedding of VE-cadherin is associated with *in vivo* angiogenesis (Reiss and Saftig, 2009). However, we cannot exclude the possibility that this observation might also be due to instability of the endothelial phenotype, as previously reported (James et al., 2010), or a consequence of sustained culture and passaging. Quantification of sprouting angiogenic potential showed that in static culture conditions (monolayer invasion assay) there was no significant difference in the number and length of sprouts between the two populations of ECs (Fig. 4E). By contrast, in engineered vessels under flow conditions, A^100^/B^5^ ECs showed a significantly higher number and length of sprouts than A^50^/B^40^ ECs (Fig. 4E). These data show that, although there are a number of functional similarities between these endothelial populations, marked differences do exist between them when placed under conditions of flow.

### Anterior and posterior ECs have different hematopoietic potential

**Fig. 5.**
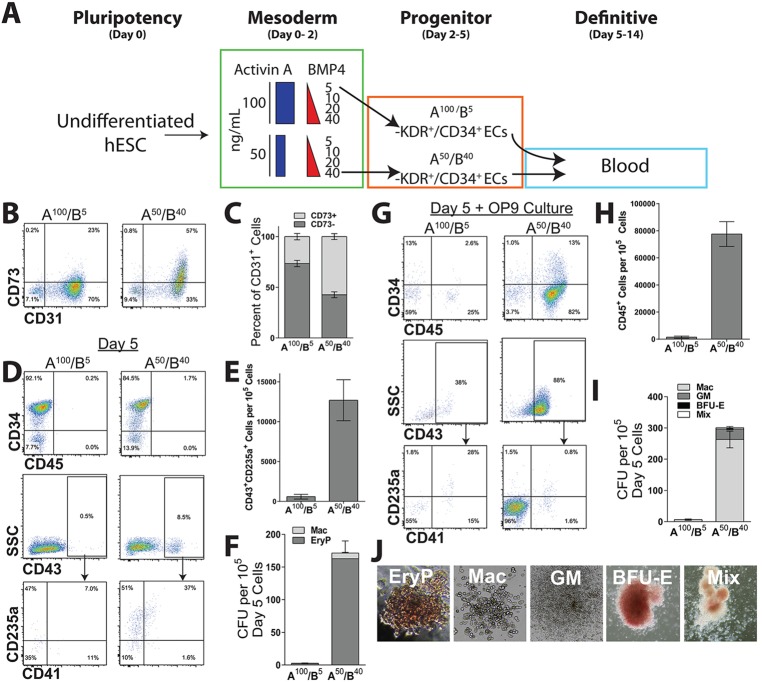
**Day 5 ECs have hematopoietic potential.** (A) The experimental approach for directing undifferentiated hESCs into A^100^/B^5^ and A^50^/B^40^ ECs followed by differentiation into blood cells. (B,C) Representative flow cytometry plots (B) and mean percentages (C) of CD73^+^ and CD73^−^ subsets of CD31^+^ A^100^/B^5^ and A^50^/B^40^ ECs (*n*=6 replicates). (D-F) Representative flow cytometry plots of hematopoietic phenotypes CD34/CD45, CD41/CD235a within the CD43 subset (D), mean numbers of cells of primitive phenotype (CD43^+^/CD235a^+^/CD41^+/−^) (*n*=6 replicates) (E), and colony-forming assays for primitive hematopoietic progenitors including primitive erythroid (EryP) and macrophage (Mac) (*n*=3 replicates) (F) generated from day 5 A^100^/B^5^ and A^50^/B^40^ ECs. (G-I) Representative flow cytometry plots of hematopoietic phenotypes CD34/CD45, CD41/CD235a within the CD43 subset (G), mean numbers of CD45^+^ hematopoietic cells generated (*n*=6 replicates) (H), and colony-forming assays for definitive erythroid/myeloid hematopoietic progenitors including macrophage (Mac), granulocyte/monocyte/macrophage (GM), large, burst-forming erythroid (BFU-E), and mixed erythroid/myeloid (Mix) colonies (*n*=3 replicates) (I) generated from 10^5^ A^100^/B^5^ ECs or A^50^/B^40^ ECs following 12-14 day secondary culture on OP9 cells. (J) Representative images for colony-forming assays. Images are magnified for EryP (100×) and Mac (50×) to show details of colony morphology.

Since a subset of endothelium (termed ‘hemogenic endothelium’) is known to give rise to hematopoietic cells in the developing embryo *in vivo* and during PSC differentiation *in vitro* (Jaffredo et al., 1998; Nishikawa et al., 1998; Lancrin et al., 2009; Choi et al., 2012; Rafii et al., 2013; Slukvin, 2013), we analyzed the blood-forming potential of A^100^/B^5^ and A^50^/B^40^ ECs (Fig. 5). Previous studies have suggested that hemogenic endothelium is confined to the portion of ECs negative for the surface marker CD73 (NT5E) (Choi et al., 2012). We found that both A^100^/B^5^ and A^50^/B^40^ ECs include a population that is CD31^+^/CD73^−^, although in differing proportions, consistent with a possible hemogenic phenotype (Fig. 5B,C).

We next examined the hematopoietic potential of A^100^/B^5^ and A^50^/B^40^ ECs over time. To determine if day 5 cells contained hematopoietic progenitors, we analyzed cells by flow cytometry for hematopoietic surface markers and by methylcellulose assays for colony-forming unit (CFU) progenitor potential. The day 5 A^50^/B^40^ EC population already contained a subset of cells expressing the earliest hematopoietic markers CD43 (SPN) and CD235a (GYPA) and, to a lesser extent, CD41 (ITGA2B) (Fig. 5D-F). This is consistent with a primitive hematopoietic phenotype (Vodyanik et al., 2006; Kennedy et al., 2012; Slukvin, 2013). By contrast, the A^100^/B^5^ ECs at day 5 lacked significant expression of CD43. As expected, neither population at this early time point exhibited significant CD45 (PTPRC), which is typically expressed later in hESC hematopoietic differentiation concomitant with broader myeloid potential (Vodyanik et al., 2006; Kennedy et al., 2012). Consistent with their primitive hematopoietic phenotype, the day 5 A^50^/B^40^ ECs generated progenitors that produced small, primitive erythroid-like (CFU-EryP) and macrophage (CFU-Mac) colonies in methylcellulose assays, whereas the A^100^/B^5^ ECs generated minimal CFU progenitors at this time point (Fig. 5F,I).

To further assay the capacity of A^100^/B^5^ and A^50^/B^40^ ECs for later erythromyeloid hematopoietic potential, day 5 cells from both populations were plated secondarily on OP9 stromal cells with hematopoietic cytokines. After 12-14 days of co-culture, cells were analyzed for hematopoietic phenotype and colony-forming progenitors. A^50^/B^40^ ECs generated a population of predominantly CD45^+^ hematopoietic cells following OP9 co-culture, a subset of which co-expressed CD34, with minimal populations expressing CD235a and/or CD41 (Fig. 5G-I). Consistent with this hematopoietic phenotype, A^50^/B^40^ ECs generated mostly myeloid CFUs consisting of both granulocyte/monocyte (GM) and macrophage (Mac) types, as well as larger erythroid burst-forming units (BFU-E) and mixed colonies containing both erythroid and myeloid elements (Mix) (Fig. 5I). By contrast, the A^100^/B^5^ ECs generated relatively limited numbers of CD45^+^ cells and few CFU progenitors (Fig. 5G-J). Interestingly, when assayed at an intermediate time point during OP9 co-culture (5 days), the A^100^/B^5^ ECs did generate a transient population of CD43^+^/CD235a^+^ cells and CFUs consisting of the primarily primitive erythroid type (supplementary material Fig. S9). Under these conditions, compared with A^100^/B^5^ ECs, A^50^/B^40^ ECs showed significantly higher levels of CD45^+^ cells, CFU-Mac and CFU-GM.

These findings suggest limited hematopoietic potential of the A^100^/B^5^ ECs, appearing later than that detected from A^50^/B^40^ ECs. This is consistent with a recent report suggesting transient hematopoietic activity of anterior mesoderm-derived endocardium during murine development (Nakano et al., 2013). Although this hematopoietic activity was detected even when the day 5 A^100^/B^5^ ECs were sorted for CD34 expression (data not shown), we cannot rule out the possibility of a hematopoietic contribution derived from a small number of less differentiated mesoderm cells among the day 5 A^100^/B^5^ ECs. Overall, however, our results suggest that hematopoietic activity is enriched within the A^50^/B^40^ relative to A^100^/B^5^ ECs, consistent with the primary origin of embryonic hematopoiesis from posterior mesoderm.

### Respecification of endothelium into cardiomyocytes by inhibition of Wnt/β-catenin signaling

**Fig. 6.**
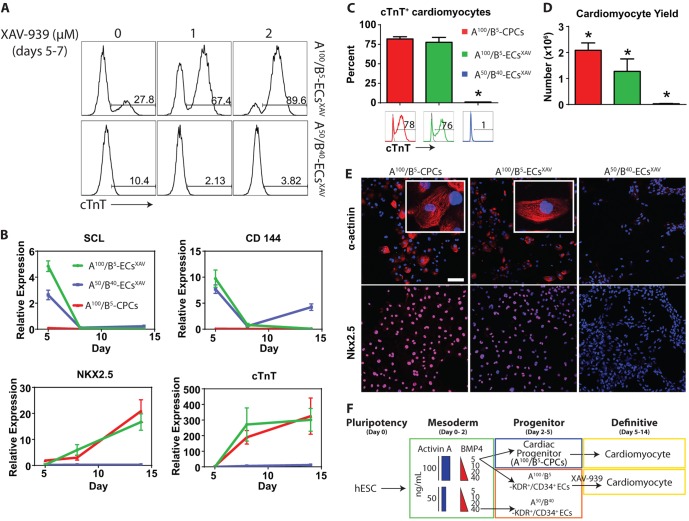
**Inhibiting Wnt/β-catenin signaling redirects A^100^/B^5^ ECs but not A^50^/B^40^ ECs into the cardiac fate with high efficiency.** (A) Flow cytometry analysis of cTnT^+^ cells from ECs treated with increasing doses of XAV-939 between days 5 and 7 of differentiation. (B) qRT-PCR analysis of *SCL*, VE-cadherin (*CD144*), *NKX2.5* and *cTnT* between differentiation days 5 and 14, comparing the lineage potential from day 5 cultures derived from A^100^/B^5^ CPCs, A^100^/B^5^ ECs^XAV^ and A^50^/B^40^ ECs^XAV^. (C) Percentage of cardiomyocytes assessed by flow cytometry for the myofilament protein cTnT among day 14 cells. (D) Cardiomyocyte yield generated from A^100^/B^5^ CPCs, A^100^/B^5^ ECs^XAV^ and A^50^/B^40^ ECs^XAV^. (E) Immunohistochemistry for α-actinin and NKX2.5 in day 14 cells generated from A^100^/B^5^ CPCs, A^100^/B^5^ ECs^XAV^ and A^50^/B^40^ ECs^XAV^. Scale bar: 100 µm. (F) Schematic illustrating that A^100^/B^5^ CPCs and A^100^/B^5^ ECs^XAV^ are lineage biased to the cardiac fate compared with A^50^/B^40^ ECs^XAV^. *n*=5-6 replicates per sample. *P<0.05 versus all other groups.

Studies have suggested that cardiac and hemogenic-endothelial lineages are closely related in the genetic and signaling mechanisms that mediate their development (Lin et al., 1998; Ferdous et al., 2009; Gekas et al., 2009; Lian et al., 2012; Van Handel et al., 2012; Chan et al., 2013; Kim et al., 2013; Palpant et al., 2013). Work from our laboratory and others indicates that activation of the Wnt/β-catenin signaling pathway is sufficient to convert cardiomyocyte progenitors into hemogenic ECs (Schoenebeck et al., 2007; Van Handel et al., 2012; Palpant et al., 2013). Based on these findings, we tested the reciprocal hypothesis that blocking Wnt/β-catenin signaling is sufficient to convert endothelium derived from our two distinct subpopulations into the cardiac lineage. hESCs were induced to differentiate using either A^100^/B^5^ or A^50^/B^40^, followed by hemogenic endothelial differentiation conditions from days 2-5 (described above). From day 5-14 of differentiation, the medium was changed to RPMI with B27 supplement, in keeping with standard cardiac differentiation methods. In the absence of Wnt/β-catenin inhibition, A^100^/B^5^ ECs showed some intrinsic capacity to convert into cardiomyocytes (Fig. 6A), whereas no cardiogenic activity was present in the A^50^/B^40^ ECs. Gene expression analysis of day 5 A^100^/B^5^ ECs suggests that the cells are primed for cardiogenesis, based on the expression of cardiac-related genes such as *GATA4* (Fig. 3E) and *MYL7* (supplementary material Fig. S4D).

To suppress Wnt/β-catenin signaling, we tested a wide range of conditions in which cells were exposed to varying doses of the small molecule tankyrase inhibitor XAV-939 (XAV) during differentiation. Strikingly, inhibition of Wnt/β-catenin signaling with 2 µM XAV between days 5 and 7 of differentiation yielded 90% beating cardiomyocytes from anterior A^100^/B^5^ ECs (Fig. 6A; supplementary material Fig. S10A). In marked contrast, inhibiting Wnt/β-catenin signaling in posterior A^50^/B^40^ ECs did not induce cardiomyocyte development (Fig. 6A; supplementary material Fig. S10B).

qPCR analysis showed that both endothelial populations, but not A^100^/B^5^ CPCs, have high-level expression of lineage markers including *SCL* and VE-cadherin on day 5 of differentiation (Fig. 6B). By day 8 (3 days post Wnt/β-catenin inhibition) these makers were significantly downregulated in ECs. By contrast, A^100^/B^5^ CPCs never expressed endothelial markers but showed appropriate activation of cardiomyocyte development on the basis of *NKX2.5* and *cTnT* expression (Fig. 6B). Among the endothelial populations, only the anterior A^100^/B^5^ ECs showed an upregulation of cardiac lineage markers after treatment with XAV, comparable to A^100^/B^5^ CPCs (Fig. 6B). Flow cytometry for cTnT^+^ cells at the day 14 time point showed that no cardiomyocytes were produced from A^50^/B^40^ ECs treated with XAV (Fig. 6C). By contrast, significant cardiomyocyte production was generated via A^100^/B^5^ CPCs and XAV-treated A^100^/B^5^ ECs, with no difference in purity between these approaches (cTnT^+^ cells generated by: A^100^/B^5^ CPCs, 81±1%; A^100^/B^5^ ECs^XAV^, 72±2%; A^50^/B^40^ ECs^XAV^, 1±0%; Fig. 6C). There was also no difference in the onset of beating during differentiation or in the intrinsic beating rate (supplementary material Movie 1 and Fig. S11A,B). Cardiomyocytes generated from XAV-treated A^100^/B^5^ ECs showed slightly lower cardiomyocyte cell yield than A^100^/B^5^ CPCs (Fig. 6D).

Immunohistochemistry showed clear myofibrillar striations in both cardiomyocyte populations on the basis of α-actinin expression as well as nuclear-localized NKX2.5 (Fig. 6E). By contrast, these cardiac markers were not detected in the XAV-treated posterior A^50^/B^40^ ECs (Fig. 6E). These findings indicate that there is a cardiogenic lineage bias among ECs and CPCs derived from A^100^/B^5^ conditions (Fig. 6F).

**Fig. 7.**
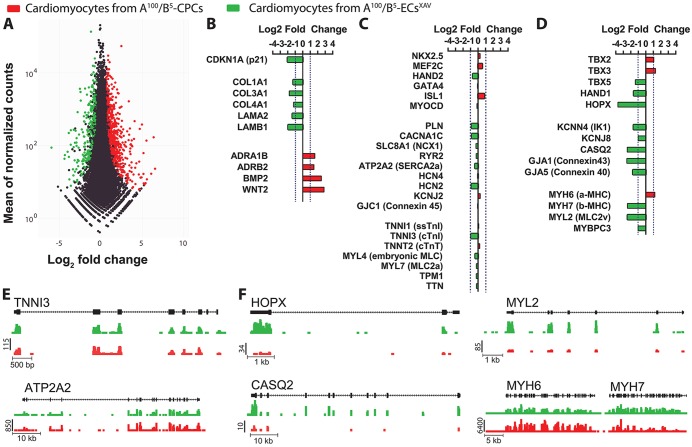
**RNA-seq analysis of cardiomyocytes generated from A^100^/B^5^ CPCs and A^100^/B^5^ ECs^XAV^.** (A) Transcripts differentially expressed with FDR<0.05 are highlighted in color: red, higher in cardiomyocytes derived from A^100^/B^5^ CPCs; green, higher in cardiomyocytes derived from A^100^/B^5^ ECs^XAV^. The subset of genes with FDR<0.05 and fold-change >2 are considered significantly different. (B-D) Hand-collated list of genes associated with cell cycle, matrix, and signaling with a greater than 2-fold difference (B), cardiac-associated genes that were not different between groups (C), and cardiac genes with a greater than 2-fold difference (D) comparing A^100^/B^5^ CPCs (red) and A^100^/B^5^ ECs^XAV^ (green). (E,F) Raw data for genes involved in cardiac development that are not different (E) or are significantly different (F) between cardiomyocytes generated from A^100^/B^5^ CPCs (red) and A^100^/B^5^ ECs^XAV^ (green). FPKM values are given.

We performed genome-wide transcriptional analysis by RNA-seq to determine similarities and differences between cardiomyocytes generated from A^100^/B^5^ CPCs and cardiomyocytes derived from XAV-treated A^100^/B^5^ ECs (Fig. 7A). These data show significant differences between the populations in the areas of extracellular matrix proteins, cell cycle regulation, and membrane signaling/glycoprotein components (Fig. 7B). Despite their origin from ECs, the XAV-treated A^100^/B^5^ ECs showed striking similarity in their cardiac gene expression profiles, including those encoding calcium-handling proteins (e.g. CACNA1C, ATP2A2), myofilament proteins (e.g. TNNI1, cTnT) and transcription factors (e.g. HAND2, GATA4, MEF2C, NKX2.5) (Fig. 7C,E; supplementary material Tables S1 and S2). Some cardiac genes were significantly different in expression between these populations and suggested a more mature ventricular phenotype in XAV-treated A^100^/B^5^ ECs compared with A^100^/B^5^ CPCs. These include genes encoding proteins such as MYL2 and MYH7, gap junction, ion channel and calcium-handling proteins (GJA1, GJA5, KCNN4, CASQ2), the cardiac maturation factor HOPX, as well as the first heart field transcription factor TBX5 (Fig. 7D,F). RNA-seq analysis of XAV-treated A^50^/B^40^ ECs indicated that, although endothelial markers were downregulated after Wnt/β-catenin inhibition (Fig. 6B), this population continued to show evidence of its endothelial-like state as indicated by the enrichment of endothelial gene ontology categories (supplementary material Fig. S5). Our data cannot distinguish epigenetic memory from a mixed population at this point.

We carried out several experiments in order to understand the nature of this cell fate conversion in greater detail. First, we tested whether cell fate conversion could occur if Wnt/β-catenin inhibition was introduced at earlier time points in differentiation, which would suggest that uncommitted mesoderm cells are giving rise to the cardiomyocytes. These results show that Wnt/β-catenin inhibition at day 3 of differentiation (the time point when XAV is added in cardiac differentiation) does not result in efficient fate conversion, arguing against residual uncommitted mesoderm cells (supplementary material Fig. S10A). Not until day 5, when the cells are fully committed hemogenic endothelium (>90% KDR^+^/CD34^+^), do we observe a robust capacity for conversion to the cardiac fate. The capacity for cell fate conversion diminishes as the cells mature toward the endothelial fate (supplementary material Fig. S10C).

Second, we were able to convert ECs to cardiomyocytes using various media, including endothelial growth medium (EGM), Stempro34 with cardiac supplement, as well as RPMI with B27 supplement (supplementary material Fig. S12). This indicates that the capacity for cell fate conversion from ECs is primarily determined by Wnt/β-catenin inhibition and not other cardiogenic factors in the media. Furthermore, we show that increasing doses of XAV do not markedly alter the number of live cells during the cell fate conversion, indicating that the cardiomyocytes generated are not the consequence of selection of a cardiogenic subset caused by the death of non-cardiogenic cells (supplementary material Fig. S11D).

Lastly, we tested by FACS whether cardiac fate conversion from endothelium was attributable solely to the CD34^+^ population, and found that cardiomyocytes were generated exclusively from the CD34^+^ population and not from the CD34^−^ population (supplementary material Fig. S11C).

## DISCUSSION

PSCs provide a promising source of human cells for therapeutic applications. Although cellular plasticity is one of the most exciting features of these cells, directing their specific fate efficiently presents a major challenge. In the current study, we show that titrating activin A and BMP4 dosage results in modulation of Wnt/β-catenin signaling activity at the onset of fate specification. This induction approach has dramatic implications for generating different populations that reflect the embryological patterning of anterior and posterior mesodermal lineages. The lineage bifurcations that occur as a consequence of the different induction signals enable the specification of progenitor populations that are capable of giving rise to high-purity definitive mesodermal derivatives such as cardiomyocytes and ECs (supplementary material Fig. S1)*.* We found variability in the efficiency of differentiation using these protocols between various hPSC lines, indicating the need for line-by-line optimization. Our approach utilizing changes in activin A and BMP4 concentration shows that these morphogens act both in a coordinated fashion and independently to orchestrate the complex aspects of lineage fate choice in mesoderm development.

Analogous to embryological events, we show that cardiac differentiation occurs most efficiently under conditions that specify an anterior-like cardiogenic mesoderm. We also show that KDR^+^/CD34^+^/VE-cadherin^+^ ECs are generated with equal potency across all conditions of activin A/BMP4 dosing. This is consistent with the observation that endothelial lineages are derived from a range of embryological origins (Wu et al., 2011; Nakano et al., 2013). Studies have shown that endocardial endothelium is derived from the anterior mesoderm in coordination with the heart-forming fields, whereas vascular endothelium involved in formation of the dorsal aorta, aorta-gonad-mesonephros (AGM) and yolk sac vasculature is generated from the posterior mesoderm (Walmsley et al., 2002; Schoenebeck et al., 2007; Misfeldt et al., 2009).

Although KDR and CD34 expression does not distinguish between these populations, we show mRNA differences in *NFATC1* and *HAND1* expression that do suggest a polarizing effect of activin A/BMP4 titration on endothelial subtypes. Our findings indicate that establishing a polarization of mesoderm at the onset of differentiation is required to specify sublineages of endothelial fate that have unique phenotype and function. Anterior ECs showed significantly higher angiogenic behavior under flow, with diminished VE-cadherin junctions at cell-cell contacts, whereas posterior ECs strongly expressed VE-cadherin with minimal sprouting under flow. EC responses to flow have been shown to occur, in part, through a junctional mechanosensory complex consisting of VE-cadherin, CD31 and KDR (Abraham et al., 2009). Although both EC populations expressed VE-cadherin, our data indicate that robust angiogenesis in anterior ECs under flow conditions is correlated with downregulation of VE-cadherin.

In addition to the molecular similarities and differences between these EC subtypes, we show unique functional characteristics of these populations in terms of their hemogenic capacity, consistent with embryonic mesodermal patterning. Compared with anterior-derived ECs, posterior-derived ECs were highly enriched in both early hematopoietic activity, as judged by surface markers, and CFU progenitors as assayed at day 5, as well as later, broader erythromyeloid activity assayed following extended OP9 co-culture with hematopoietic cytokines. These findings are consistent with the embryonic origin of most hematopoietic precursors from posterior mesoderm. Interestingly, we also observed limited, transient hematopoietic potential from anterior-derived ECs. Although we cannot rule out contamination from a small population of undifferentiated cells giving rise to these hematopoietic progeny under hematopoiesis-inducing conditions during OP9 co-culture, this finding could also be consistent with a recent study showing transient hematopoietic potential from cells of endocardial (anterior mesoderm) origin (Nakano et al., 2013). Furthermore, the primarily primitive hematopoietic phenotype observed from anterior-like cells is consistent with relatively higher activin/nodal and lower Wnt/β-catenin signaling during the mesodermal patterning of these cells, as recently shown to be favorable for primitive hematopoiesis (Kennedy et al., 2012; Sturgeon et al., 2014).

Our laboratory and others have shown that a failure to express key molecules involved in fate decision in the developing endothelium (e.g. TMEM88, SCL or Cloche) leads to the interconversion of cells between the cardiac and endothelial fates at least in part through regulation of Wnt/β-catenin signaling (Schoenebeck et al., 2007; Van Handel et al., 2012; Palpant et al., 2013). This study shows that generating ECs from prepatterned mesoderm results in a marked cardiogenic lineage bias only in anterior-derived ECs. In particular, we show that transient antagonism of Wnt/β-catenin signaling facilitates the conversion of endothelium derived from anterior mesoderm into cardiomyocytes. By contrast, endothelium derived from posterior mesoderm showed no capacity for cardiogenic lineage conversion after Wnt/β-catenin inhibition. More definitive assays in the future should include *in vitro* lineage-tracing studies to confirm these observations.

The capacity to direct the differentiation of high-purity progenitor and definitive cell types from hPSCs is a critical step toward understanding developmental mechanisms, disease etiology and generating therapeutically relevant cell preparations. This study shows that activin A and BMP4 are sufficient to direct the polarization of mesoderm *in vitro*, which has marked consequences for efficient cell fate specification into cardiac, endothelial and hematopoietic lineages.

## MATERIALS AND METHODS

### Cell culture

Human ESCs and iPSCs were cultured on Matrigel (BD Biosciences) coated plates and maintained in an undifferentiated state with either mouse embryonic fibroblast (MEF)-conditioned media containing 5 ng/ml bFGF (Peprotech, 100-18B) (for RUES2 hESCs, IMR90 hiPSCs, VN1 hiPSCs) or defined media including X-Vivo (Lonza) (for ELF1 hESCs), or mTESR (Stem Cell Technologies) (for RUES2 hESCs, WTC11 hiPSCs). A subset of experiments was performed using RUES2 hESCs modified by lentivirus to express the β-catenin-activated reporter BAR-Venus, as we described previously (Palpant et al., 2013).

### hESC directed differentiation

Standard cardiomyocyte directed differentiation using a monolayer platform was performed with a modified protocol based on previous reports (Laflamme et al., 2007; Paige et al., 2010; Lian et al., 2012). Endothelial differentiation involved initiation of differentiation with activin A and BMP4. On day 2, the medium was changed to a composition adapted from work reported previously (Kennedy et al., 2007): Stempro34 (Invitrogen, 10640019) containing 200 ng/ml VEGF (Peprotech, 100-20), 5 ng/ml bFGF (Peprotech, 100-18B), 10 ng/ml BMP4 (R&D Systems, 314-BP-050), 0.4 mM monothioglycerol, 50 µg/ml ascorbic acid, 2 mM L-glutamine (Invitrogen, 25030-081) and Pen-Strep (Invitrogen, 15140-163). Medium was not changed until day 5. For additional information, see the supplementary Materials and Methods and Fig. S6.

### Colony-forming assays and hematopoietic differentiation on OP9 cells

OP9 feeder cells were seeded in 24-well plates the day prior to co-culture. Day 5 unsorted or CD34^+^ sorted hESC-derived ECs were seeded onto OP9 cells at 1×10^5^ cells per well in alpha-MEM (Invitrogen) with 10% FBS (Hyclone), Pen-Strep (Invitrogen) and recombinant cytokines. Colonies were scored by morphology after 12-14 days as small, primitive erythroid (CFU-EryP), macrophage (CFU-Mac), granulocyte/monocyte/macrophage (CFU-GM), large, burst-forming erythroid (BFU-E), or mixed colonies containing both erythroid and myeloid elements (CFU-Mix). For details, see the supplementary Materials and Methods.

### Endothelial cell differentiation and analysis

Unsorted or CD34^+^ sorted anterior- or posterior-derived ECs were plated in gelatin-coated tissue culture flasks with EGM media (Lonza, CC-3124) containing 20 ng/ml VEGF (Peprotech, 100-20), 20 ng/ml bFGF (Peprotech, 100-18B) and 1 µM CHIR-99021 (Cayman Chemical, 13122). Cells were maintained until day 14, at which point cells were isolated, analyzed by flow cytometry for CD31 expression, and then assessed in assays for tube formation, angiogenesis, and fluid shear stress in microfluidic channels as described in the supplementary Materials and Methods.

### Immunofluorescence

Cells were fixed, stained with primary antibodies then Alexa Fluor-conjugated secondary antibodies (Invitrogen), and counterstained with DAPI or Hoechst 33342 as described in the supplementary Materials and Methods.

### Quantitative (q) RT-PCR

Total RNA was isolated (from unsorted cell populations, unless specified otherwise) using the RNeasy Miniprep Kit (Qiagen) and first-strand cDNA synthesized using the Superscript III Reverse Transcriptase Kit (Invitrogen). qPCR was performed using the Sensimix SYBR PCR Kit (Bioline) on a 7900HT fast real-time PCR system (Applied Biosystems). The copy number for each transcript is expressed relative to *HPRT*. Primers are listed in supplementary material Table S3. 

### Flow cytometry

BAR-Venus RUES2 cells were analyzed for intrinsic Venus fluorescence by FACS. Wild-type RUES2 cells were labeled for flow cytometry using antibodies as described in the supplementary Materials and Methods. Cells were analyzed using a FACSCANTO II or sorted on a FACSARIA II with FACSDiva software (BD Biosciences). Instrument settings were adjusted to avoid spectral overlap. Data analysis was performed using FlowJo (Tree Star). For further details of FACS and Wnt/β-catenin signaling analysis using BAR-Venus see the supplementary Materials and Methods.

### Proteomics

Peptides were isolated from cells and measured by nano-LC-MS/MS on a Q Exactive (Thermo Scientific) equipped with a NanoAcquity system (Waters) as described in the supplementary Materials and Methods. Identification and label-free quantification of peptides were performed with MaxQuant 1.3.0.5 using a 1% false discovery rate (FDR) against the human Swiss-Prot/TrEMB database downloaded from Uniprot on October 11th, 2013. For further details see the supplementary Materials and Methods.

### RNA-seq

Samples were submitted to University of Washington High-Throughput Genomic Sequencing Center for isolation and analysis. RNA-seq was performed on poly-A-enriched samples using Illumina TruSeq. Differentially expressed genes were classified according to gene ontology using the NIAID Database for Annotation, Visualization and Integrated Discovery (DAVID/EASE, http://david.abcc.ncifcrf.gov/). RNA-seq data have been deposited in the NCBI Gene Expression Omnibus database with accession number GSE55275. Separately, FPKM (fragments per kilobase of exon per million fragments mapped) values were computed using Cufflinks version 2.1.1 (Trapnell et al., 2010) and UCSC gene annotations. For additional information, see the supplementary Materials and Methods.

### Statistics

Single variable analysis between two samples was compared by Student's *t*-test. Single and multivariable assays were analyzed by one-way or two-way ANOVA. Results are presented as mean±s.e.m. *P*<0.05 was considered significant.

## Supplementary Material

Supplementary information
