# Supplementary material for: Inhibition of β-catenin signaling respecifies anterior-like endothelium into beating human cardiomyocytes
Source: Development. 2015 Sep 15;142(18):3198–209. doi: 10.1242/dev.117010 (PMC4582173; doi:10.1242/dev.117010)
Supplement: Supplementary information [file supp_142_18_3198__index.html]

Supplementary information 

# Inhibition of β-catenin signaling respecifies anterior-like endothelium into beating human cardiomyocytes

## DEV117010 Supplementary information

**Files in this Data Supplement:**

- Supplementary information
