# Supplementary material for: Inhibition of β-catenin signaling respecifies anterior-like endothelium into beating human cardiomyocytes
Source: Development. 2015 Sep 15;142(18):3198–209. doi: 10.1242/dev.117010 (PMC4582173; doi:10.1242/dev.117010)
Supplement: Supplementary information [file supp_dev.117010_DEV117010supp.pdf]

Supplementary Materials and Methods

Human ESC Wnt/β-catenin signaling analysis. WT RUES2 human embryonic stem cells were used in this study. A subset of experiments were performed using RUES2 hESCs modified by lentivirus to express the β-catenin activated reporter (BAR-Venus) as we have described previously (Palpant et al., 2013). The BAR-Venus reporter in combination with gene expression analysis provides insight into the activity of the Wnt/β-catenin pathway in the context of differentiation. BAR-Venus activity was measured by FACS analysis from bulk cell populations and calculated on a log10 scale.

Human ESC Cardiac Directed Differentiation. Standard cardiomyocyte directed differentiation using a monolayer platform was performed with a modified protocol based on previous reports (Laflamme et al., 2007; Paige et al., 2010; Lian et al., 2012). The differentiation set up was initiated by plating undifferentiated hESCs as single cells as described previously (Gantz et al., 2012; Palpant et al., 2013). The cultures were treated with CHIR-99021 (Cayman chemical, 13122) for 24 hours before reaching confluence. In initial experiments, cells were induced to differentiate (designated day 0) by replacing the culturing media with RPMI media (Invitrogen, 11875-119) containing different doses of activin A (R&D Systems, 338-AC-050) (50 or 100 ng/ml), 1:60 diluted Matrigel (BD), and insulin-free B27 supplement (Invitrogen, 0050129SA). An RPMI media change the following day (17 hours) included different doses of BMP4 (R&D Systems, 314-BP-050) (5, 10, 20 or 40 ng/ml), 1 uM CHIR-99021, and insulin-free B27 supplement. CHIR-99021 is added during this step to reinforce signaling through the Wnt/β-catenin signaling pathway for efficient specification of mesoderm (Paige et al., 2010; Lian et al., 2012; Paige et al., 2012; Palpant et al., 2013). In a subset of experiments, 1 uM XAV-939 (Tocris, 3748) was included at day 3 of differentiation. XAV-939 is added in this step of the protocol to reinforce endogenous

Development • Supplementary information

inhibitors of the Wnt/β-catenin signaling pathway for efficient specification of the cardiac lineage (Paige et al., 2010; Willems et al., 2011; Lian et al., 2012; Palpant et al., 2013). RPMI containing insulin-free B27 supplement was utilized until differentiation day 7 in which the media is replaced with RPMI containing a B27 supplement that includes insulin (Invitrogen, 17504044). Subsequent media changes included the insulin-containing supplement. Comparison of differentiation efficiency was analyzed using this protocol with various hiPSC and hESC lines (supplementary material Fig. S6).

Human ESC Endothelial Differentiation. Endothelial differentiation involved initiation of differentiation with activin A and BMP4 as described above. On day 2 media is changed to media adapted from work reported previously (Kennedy et al., 2007): Stempro34 (Invitrogen, 10640019) backbone media containing 200 ng/ml VEGF (Peprotech, 100-20), 5 ng/ml bFGF (Peprotech, 100-18B), 10 ng/ml BMP4 (R&D Systems, 314-BP-050), $4x10^{-4}$ M monothioglycerol, 50 ug/ml ascorbic acid, 2 mM L-glutamine (Invitrogen, 25030-081), and pen-strep (Invitrogen, 15140-163) . Media was not changed until day 5. For cardiac differentiation from endothelium, day 5 cells were exposed to 2 uM XAV-939 in RPMI media containing B27 supplement without insulin and this was changed on day 7 – 14 to RPMI media containing B27 supplement with insulin. For endothelial maturation from day 5 endothelium, cells were re-plated on gelatin-coated plates in EGM (Lonza) containing 20 ng/ml VEGF (R&D Systems, FAB357P), 20 ng/ml bFGF, and 1 uM CHIR-99021. Since A100/B5-ECs were susceptible to sheer stress induced cell death, this protocol was modified for analysis of FACS sorted populations as follows: on day 5 cells were pretreated for 2 h with Stempro34 with Y-27632. Cells were harvested and labeled with CD34 antibody (BD Biosciences, 340430) and then FACS sorted. Cells were re-plated in EGM with Y-27632 for 2 days and thereafter with RPMI containing Y 27632 and B27 supplement with insulin.

Comparison of differentiation efficiency was analyzed using this protocol with various hiPSC and hESC lines (supplementary material Fig. S6b-d).

Colony forming assays and hematopoietic differentiation on OP9 Cells. OP9 feeder cells were seeded in 24-well plates the day prior to co-culture. Day 5 unsorted or $CD34^+$ sorted hESC-derived endothelial cells were seeded onto OP9 cells at $1x10^5$ cells per well in alpha-MEM (Invitrogen) with 10% FBS (Hyclone), penicillin/streptomycin (Invitrogen), and recombinant cytokines (from R&D Systems): hSCF (50 ng/ml), hTPO (20 ng/ml), hIL-6 (20 ng/ml), hIL-3 (20 ng/ml), hFLT3L (20 ng/ml). Following 6 or 12-14 days of co-culture, non-adherent/loosely adherent cells were removed by pipetting and passed through a 35 µm cell strainer (BD Falcon) and analyzed by flow cytometry. Day 5 hESC-derived endothelial cells or cells harvested following OP9 co-culture were plated for colony forming-unit (CFU) progenitors in methylcellulose containing human cytokines (H4034, Stem Cell Technologies). Colonies were scored by morphology after 12-14 days as small, primitive erythroid (CFU-EryP), macrophage (CFU-Mac), granulocyte/monocyte/macrophage (CFU-GM), large, burst-forming erythroid (BFU-E), or mixed (CFU-Mix) colonies containing both erythroid and myeloid elements.

Endothelial Cell Differentiation and Analysis. Unsorted or $CD34^+$ sorted anterior and posterior-derived hemogenic endothelial cells were plated in gelatin-coated tissue culture flasks with EGM media (Lonza, CC-3124) containing 20 ng/ml VEGF (Peprotech, 100-20), 20 ng/ml bFGF (Peprotech, 100-18B), and 1 uM CHIR-99021 (Cayman Chemical, 13122). Cells were maintained until day 14 at which point cells were isolated, analyzed by flow cytometry for CD31 expression, and cells then applied to secondary assays. For 2D endothelial marker analysis, cells were grown to confluency overnight after re-plating at $100K/cm^2$ on 0.2% gelatin-coated coverslips. Cells were then fixed for 10 minutes in 3.7%

Development • Supplementary information

formaldehyde, washed in PBS, and stained for CD31, Ki67, and Von Willebrand Factor. Images were acquired with a Nikon TiE inverted widefield fluorescence microscope with 40X objective and 1392x1040 resolution. Proliferation was quantified by determining the percentage of Ki67-positive cells in anterior and posterior populations (n: biological replicates (# of scored cells) = 3(302) and 3(246) respectively). **Tube formation assay:** Type I collagen extracted from rat tails as previously described (Zheng et al., 2012) was dissolved in 0.1% acetic acid at 15 mg/ml and stored at 4°C. Before use, collagen gel was neutralized and diluted with 1 M NaOH, EGM, and 10X M199 (Sigma M0650). Gel was then mixed with day 14 cells at a density of $2x10^6$/ml (final collagen concentration: 2 mg/ml), pipetted into 4 mm diameter well (10 ul volume) of angiogenesis μ slides (Ibidi), and allowed to gel for 20 minutes at 37°C. Cultures were fed with EGM supplemented with 20 ng/ml VEGF, 20 ng/ml bFGF, and 1 uM Chiron (EGM+factors) for 2 days, fixed in 3.7% formaldehyde for 10 min, washed in PBS, and stained for CD31 and phalloidin. **Angiogenesis assay:** Day 14 endothelial cells were seeded on top of acellular 2 mg/ml collagen disks (4mm D x 2mm H) at a density of 400 cells/mm^2 and fed with EGM+factors for 3 days. Samples were fixed for 10 minutes in 3.7% formaldehyde, washed in PBS, and stained for phalloidin and CD31. Three dimensional z-stack images of both angiogenesis and tube formation assays were acquired on a Nikon A1R confocal microscope with 20X objective, 1024x1024 resolution. Maximum intensity projections and orthogonal views were obtained using Fiji software. Angiogenic sprouting was quantified by manually counting the number of sprouts extending downward from the monolayer and normalizing to the cross sectional area of the field of view. The average length of sprouts was determined using Fiji measure function (n: 3 biological replicates). **Engineered Microvessels:** Microfluidic channels were created in collagen gel using an injection molding and soft lithographic technique as described previously (Zheng et al., 2012). Following fabrication, day 14 cells were seeded in the

Development • Supplementary information

conduit at a density of $10x10^6$/ml and a volume of 10 µl and allowed to circumferentially attach for 3 hours in static conditions. After endothelial cell attachment in the channel, microvessels are cultured under gravity driven flow by replacing media (EGM+factors) at the inlet every 12 hours for 4 days. Microvessels were then fixed by perfusing 3.7% formaldehyde for 20 minutes, washed in PBS, then stained for CD31, VE-cadherin, and Von Willebrand Factor. Three dimensional z-stack images were acquired on a Nikon A1R confocal microscope with 10X objective, 512x512 resolution. Maximum intensity projections and orthogonal views were obtained using Fiji software. The number of angiogenic sprouts and average length were quantitated in the same way as the semi-3D angiogenesis assay (n: 3 biological replicates). The number of sprouts was normalized to the surface area of the lumen. **Immunofluorescence staining:** Following fixation, samples were blocked in 2% BSA and 0.5% Triton X-100 in PBS for 1 h followed by an overnight incubation with primary antibodies (Rb pAb to hCD31 (Abcam 28364), Ms pAb to VE-cadherin (Abcam 7047), or Rb pAb to Ki67 (Abcam 16667)). Samples were washed with PBS and incubated with secondary antibodies for 1 h at room temperature (Alexa Fluor 488 Phalloidin (Invitrogen A12379), Sh pAb to Von Willebrand Factor (Abcam 8822), Alexa Fluor goat anti-mouse 647 (Invitrogen A21235), Alexa Fluor goat anti-rabbit 568 (Invitrogen A11011)) and counterstained with Hoechst 33342 (Sigma B2261). Following secondary incubation, samples were washed in PBS. Tube formation assay, angiogenesis assay, and microvessels were kept in PBS until imaging. Coverslips were mounted face down onto slides with Aqua Poly Mount (Polysciences 18606).

Flow Cytometry. BAR-venus RUES2 cells were analyzed for intrinsic Venus fluorescence by FACS. WT RUES2 cells were labeled for flow cytometry using the following antibodies: human PDGFRα APC (R&D Systems) and human VEGFR2/KDR PE (R&D Systems),

Development • Supplementary information

CD31 (BD Biosciences, 555445), CD144 (VE-cadherin) (E-biosciences, 17-1449-42), CD34 (BD Biosciences, 340430), CD73 (BD Pharmingen), and cardiac troponin T (Pierce, MA5-12960) or corresponding isotype controls. For hematopoietic analysis, cells were stained with various combinations of the following monoclonal antibodies for cell surface analysis: APC-conjugated anti-human CD43 (Clone 1G10, BD Pharmingen), PE-conjugated anti-human CD235a (Clone GA-R2, BD Pharmingen), PECy7-conjugated anti-human CD45 (Clone H130, Biolegend), FITC-conjugated anti-human CD34 (BD Pharmingen), and PECy7-conjugated anti-human CD41 (Clone HIP8, Biolegend), or corresponding isotype control antibodies. DAPI was used to exclude dead cells. Cells were analyzed using a BD FACSCANTO II or sorted on a BD FACSARIA II (Beckton Dickinson, San Jose, CA) with FACSDiva software (BD Biosciences). Instrument settings were adjusted to avoid spectral overlap. Data analysis was performed using FlowJo (Tree Star, Ashland, Oregon). For the live/dead assay cells were isolated and stained with calcein AM and EthD-1 using the LIVE/DEAD viability/cytotoxicity kit for mammalian cells (Molecular Probes). Cells were analyzed by flow cytometry using FITC to detect live cells and Texas Red to detect dead cells.

Immunofluorescence. Cells were fixed with either 4% paraformaldehyde or methanol, permeabilized in PBS containing 0.025% Triton-X, and blocked in PBS containing 1.5% normal goat serum. Cells were stained with NKX2.5 (R&D Systems, Cat.# AF2444, 1:400), alpha-actinin (Sigma, Clone EA-53; Cat.#A7811, 1:800), CD31 (Dako, Clone JC/70A Cat.# M0823, 1:10), SM-MHC (Sigma, Clone HSM-V Cat.# M7786, 1:100), SM-Actin (Dako, Clone 1A4, Cat.#M0851, 1:500) followed by secondary staining with Alexa Fluor-594 donkey anti-goat (Invitrogen lot #1180089, 1:200) or Alexa Fluor-594 goat anti-mouse

Development • Supplementary information

(Invitrogen lot # 1219862, 1:200). Nuclei were counterstained with DAPI. Human fetal heart and canine heart sections were used positive controls.

Proteomics: *Sample preparation for mass spectrometry:* Cells were washed in 1xPBS and frozen at -20°C. Cells were lysed in 1 M urea, 50 mM ammonium bicarbonate pH 7.5, and heated to 60°C for 30 min. Cell debris was removed by centrifugation (10000 rpm, 30 min). Protein was quantified and normalized using a BCA assay and then reduced with 2 mM DTT for 15 min at 56°C, alkylated with 15 mM iodoacetamide for 15 min in the dark at 37°C, and digested overnight with a 1:50 ratio of trypsin to total protein. The resulting peptides were acidified with 50% formic acid, 10% trifluoroacetic acid and desalted on Waters Sep-Pak C18 cartridges. After desalting, samples were dried under vacuum in a SpeedVac (Eppendorf Vacufuge) and resolved in 50 µl 1% acetonitrile, 0.1% formic acid.

Nano-LC-MS/MS measurements: Peptides were measured by nano-LC-MS/MS on a Q Exactive (Thermo Scientific) equipped with a NanoAcquity system (Waters). Peptides were trapped on a 30cm IntegraFrit column (75mm ID packed with 2cm Magic C18 AQ 5 µM /100 Å beads) at a flow rate of 2 µl/min. Peptides were separated online by reverse phase chromatography using a heated 50°C home-made 28 cm C18 column (75 mm ID packed with Magic C18 AQ 5 µM /100 Å beads) in a 120 min gradient (1% to 45% acetonitrile with 0.1% formic acid) with a flow rate of 250 nl/min. Data-dependent acquisition was conducted on the Q Exactive with the following settings: 70,000 resolution, 350-2000 m/z full scan, Top 10, and an 1.8 m/z isolation window.

Data Processing and Analysis: Identification and label free quantification of peptides were done with MaxQuant 1.3.0.5 using a 1% false discovery rate (FDR) against the human Swiss-Prot/TrEMB database downloaded from Uniprot on October 11th, 2013. Three replicates were analyzed per condition. Peptides were searched for variable modification of

Development • Supplementary information

n-term protein acetylation, oxidation (M), deamidation (NQ), with a 6 ppm mass error and a match between run window of 4 min. Forward and reverse human sequences as well as common contaminants were removed during analysis in Perseus 1.4.1.3. Conditions were compared using principle component analysis and unsupervised hierarchical clustering. Proteins that were significantly regulated between conditions were identified using a permutation-based t-test (S0, FDR 5%).

RNA-seq. Total RNA was isolated with RNALater (Qiagen, 76104). Unsorted cell populations were isolated for RNA-seq. For each group, 2 biological replicates were submitted for analysis. Samples were submitted to University of Washington High Throughput Genomic Sequencing Center for isolation and analysis. RNA-seq was performed on poly-A enriched samples using Illumina TruSeq. Differentially expressed genes were classified according to gene ontology using the NIAID Database for Annotation, Visualization and Integrated Discovery (DAVID/EASE, http://david.abcc.ncifcrf.gov/). The RNA-seq data have been deposited in the NCBI Gene Expression Omnibus database. The GEO accession number is GSE55275. Reads were aligned with Gsnap version 2013-11-27 (Wu and Nacu, 2010) to version hg19 of the human genome sequence obtained from the UCSC Genome Browser (Karolchik et al., 2014). Strand-specific read counts were generated against gene annotations taken from Ensembl release 72 (Flicek et al., 2014). Reads with more than one reported alignment were not counted. Differential expression was evaluated using DESeq version 1.12.1 (Anders and Huber, 2010). Genes with an FDR adjusted p-value (i.e. "q-value") of less than 0.05 were classified as differentially expressed. Separately, FPKM values were computed using Cufflinks version 2.1.1 (Trapnell et al., 2010) and UCSC gene annotations.

Development • Supplementary information

Supplementary references

Anders, S. and Huber, W. (2010). Differential expression analysis for sequence count data. *Genome Biol.* **11**, R106.

Flicek, P., Amode, M. R., Barrell, D., Beal, K., Billis, K., Brent, S., Carvalho-Silva, D., Clapham, P., Coates, G., Fitzgerald, S. et al. (2014). Ensembl 2014. *Nucleic Acids Res.* **42**, D749-D755.

Gantz, J. A., Palpant, N. J., Welikson, R. E., Hauschka, S. D., Murry, C. E. and Laflamme, M. A. (2012). Targeted genomic integration of a selectable floxed dual fluorescence reporter in human embryonic stem cells. *PLoS ONE* **7**, e46971.

Karolchik, D., Barber, G. P., Casper, J., Clawson, H., Cline, M. S., Diekhans, M., Dreszer, T. R., Fujita, P. A., Guruvadoo, L., Haeussler, M. et al. (2014). The UCSC Genome Browser database: 2014 update. *Nucleic Acids Res.* **42**, D764-D770.

Willems, E., Spiering, S., Davidovics, H., Lanier, M., Xia, Z., Dawson, M., Cashman, J. and Mercola, M. (2011). Small-molecule inhibitors of the Wnt pathway potently promote cardiomyocytes from human embryonic stem cell-derived mesoderm. *Circ. Res.* **109**, 360-364.

Wu, T. D. and Nacu, S. (2010). Fast and SNP-tolerant detection of complex variants and splicing in short reads. *Bioinformatics* **26**, 873-881.

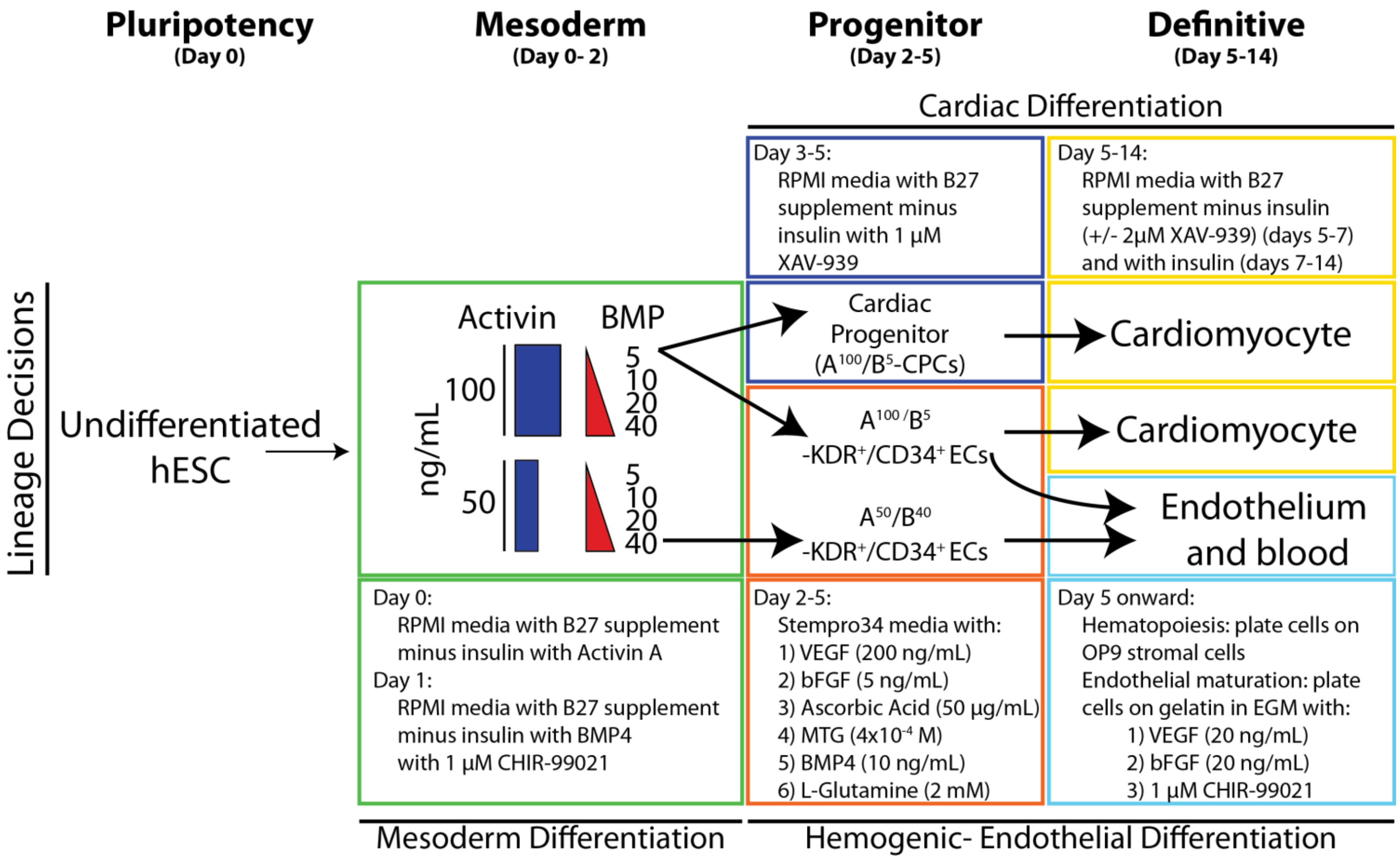

Supplemental Figure 1. Outline of conditions for specification of cell fate transitions in mesoderm differentiation from hPSCs. Schematic description of cell states and protocols for directed differentiation from human pluripotent stem cells into mesoderm specified by different concetrations of Activin A and BMP4. From mesoderm, Activin $A^{100}/BMP4^{5}$ is optimal for generating cardiac progenitor cells (A^{100}/B^{5}-CPCs) and mature cardiomyocytes. Alternatively, cells can be directed into endothelial cells generated under Activin $A^{100}/BMP4^{5}$ (A^{100}/B^{5}– $KDR^{+}/CD34^{+}$ cells) or Activin $A^{50}/BMP4^{40}$ (A^{50}/B^{40}– $KDR^{+}/CD34^{+}$ cells). Endothelium can be further differentiated into functional blood and endothelial cells. Using XAV to inhibit Wnt signaling, A^{100}/B^{5}– $KDR^{+}/CD34^{+}$ cells but not A^{50}/B^{40}– $KDR^{+}/CD34^{+}$ cells can be re-directed into cardiomyocytes similar to A^{100}/B^{5}-CPCs.

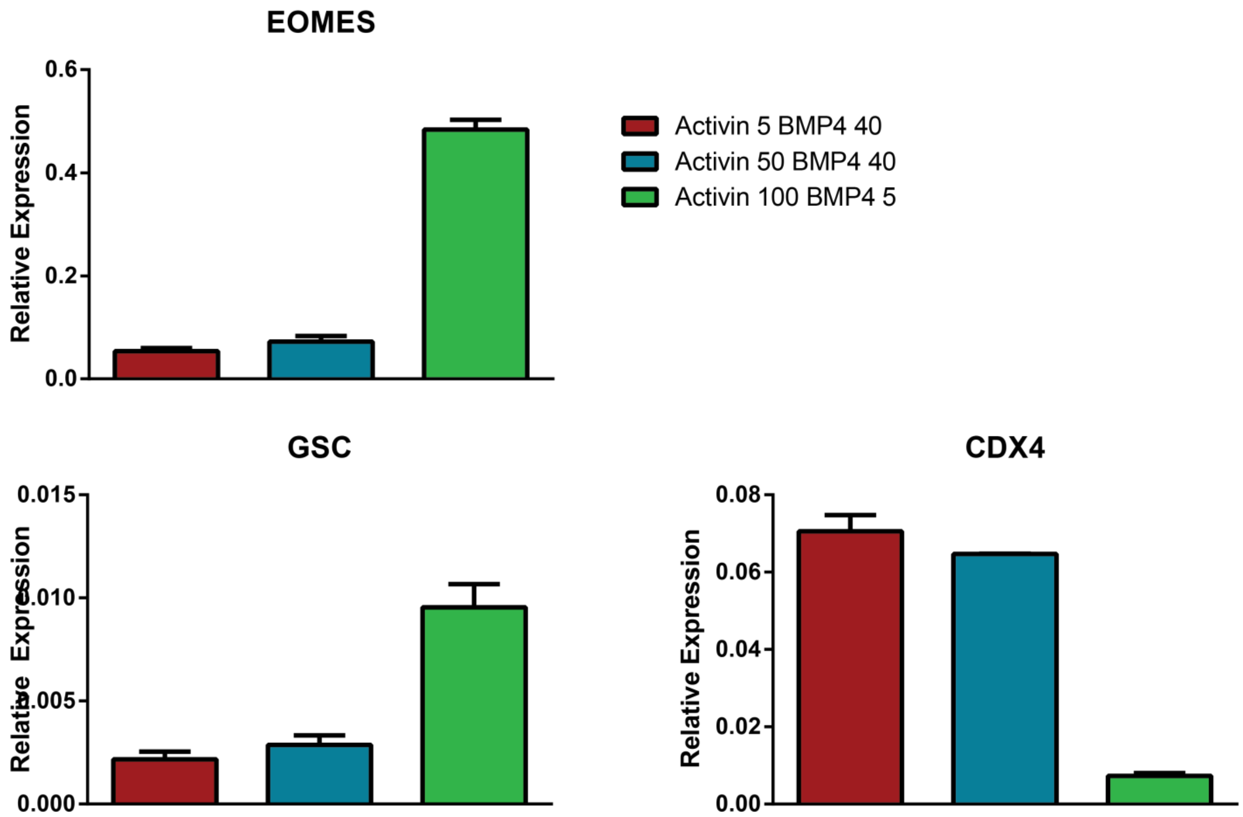

Supplemental Figure 2. Effect of Activin A and BMP4 dosing on the expression of anterior vs. posterior mesodermal markers. Cells were induced for differentiation using either A^{5}/B^{40}, A^{50}/B^{40}, or A^{100}/B^{5} to determine if varying the dose of Activin A had an effect on posterior mesoderm gene expression. These data show that there is no significant difference in anterior markers EOMES and GSC as well as the posterior marker CDX4 indicating that under these differentiation conditions the most significant change in patterning occurs in the shift from 100 ng/mL Activin A to 50 ng/mL Activin A.

Development • Supplementary information

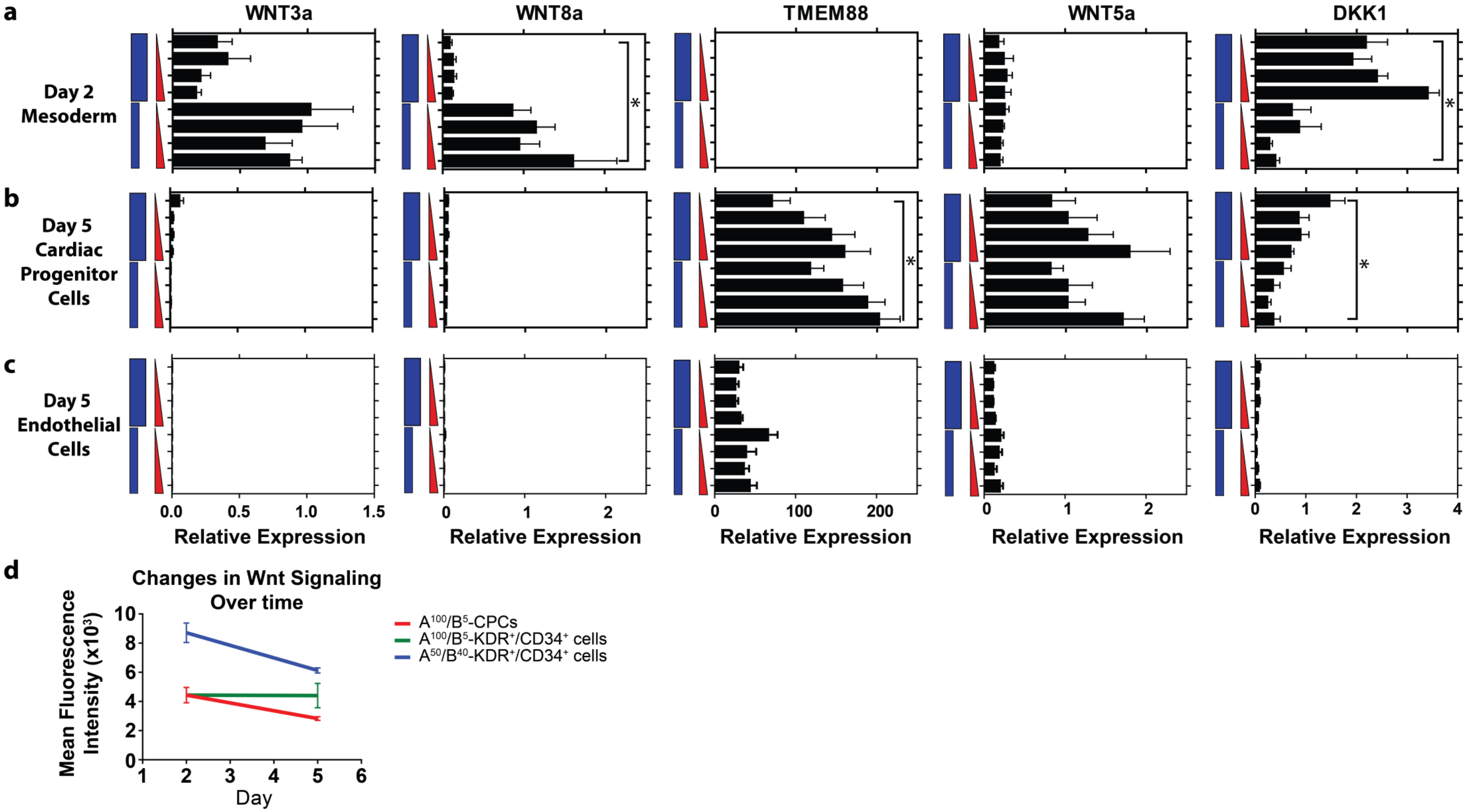

Supplemental Figure 3. Modulators of Wnt/β-catenin signaling titrate pathway activity during directed differentiation of mesoderm derivatives. (a-c) Quantitative PCR analysis of expression levels for Wnt ligands Wnt3a and Wnt8a as well as inhibitors including TMEM88, Wnt5a, and Dkk1 at different time points of differentiation including day 2 specification of mesoderm (a), day 5 differentiation into the cardiac progenitor cells (CPCs) (b) and day 5 differentiation into KDR^+CD34^+ cells (c) across different doses of Activin A and BMP4. (d) Changes in BAR-Venus activity across time derived from data presented in Figure 1c, 2c, and 3d. n=5 biological replicates per sample. * $P < 0.05$.

Development • Supplementary information

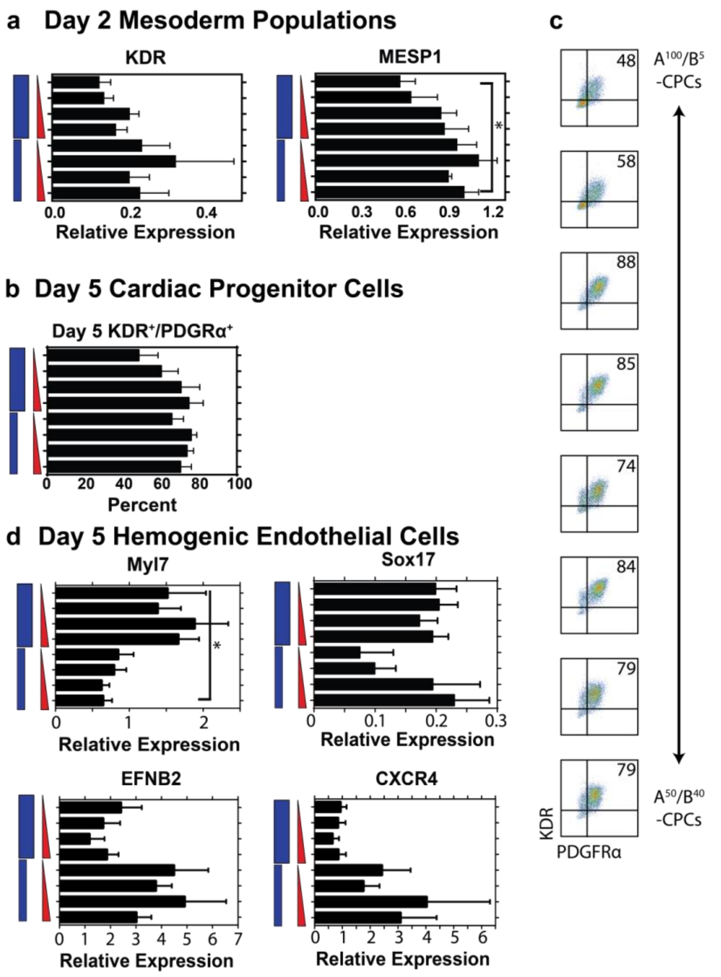

Supplemental Figure 4. Molecular analysis of anterior/posterior mesoderm formation. (a) Markers of mesoderm formation generated by different induction approaches were analyzed at day 2 of differentiation by quantitative RT-PCR including KDR and MESP1. (b-c) Day 5 cardiac progenitor cells induced under different mesodermal patterning conditions were analyzed by flow cytometry for KDR and PDGFRα. Mean data are shown for 5 biological replicates (b) and representative flow plots are shown in (c). (d) Quantitative RT-PCR for various markers at day 5 of endothelial differentiation based on different mesoderm induction approaches including the cardiac marker (MYL7) as well as endothelial markers SOX17, EFNB2, and CXCR4. n = 5 biological replicates for each experiment. * $P < 0.05$.

Development • Supplementary information

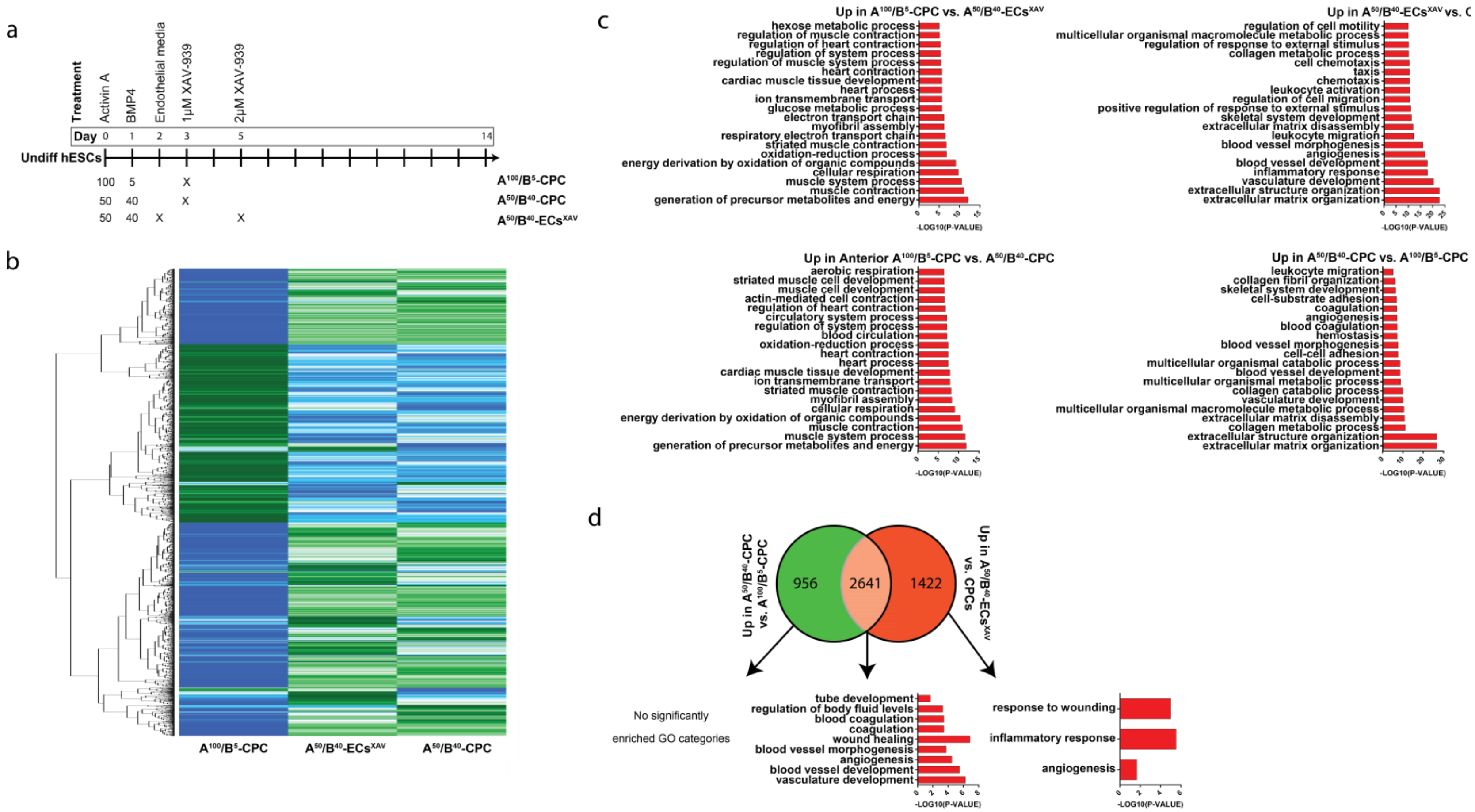

Supplemental Figure 5. RNA-seq of cells at day 14 of differentiation under various treatment regimens. (a) Schematic diagram of differentiation protocols tested. These include standard cardiac differentiation (A^{100}/B^{5}-CPC), cardiac differentiation from posterior-like mesoderm (A^{50}/B^{40}-CPC), and Wnt inhibition from day 5 posterior hemogenic endothelium (A^{50}/B^{40}-ECs^{XAV}). All samples were carried out to day 14 at which point cells were isolated and submitted for RNA-seq analysis. (b) Heat map analysis of the mean data from biological replicates for each of the assays showing that despite differentiation conditions, cells from posterior mesoderm share a similar transcriptional profile compared to cardiomyocytes. (c) Gene ontology analysis of gene sets enriched in different comparisons as described in (a). Cardiac gene ontologies are highly enriched in cardiac differentiation compared to endothelial lineage programs that are robustly enriched in lineages derived from posterior-like mesodermal origins. (d) Comparison of the genes commonly shared between cell types generated from posterior-like mesoderm show enrichment of genes involved in endothelial and blood development.

Development • Supplementary information

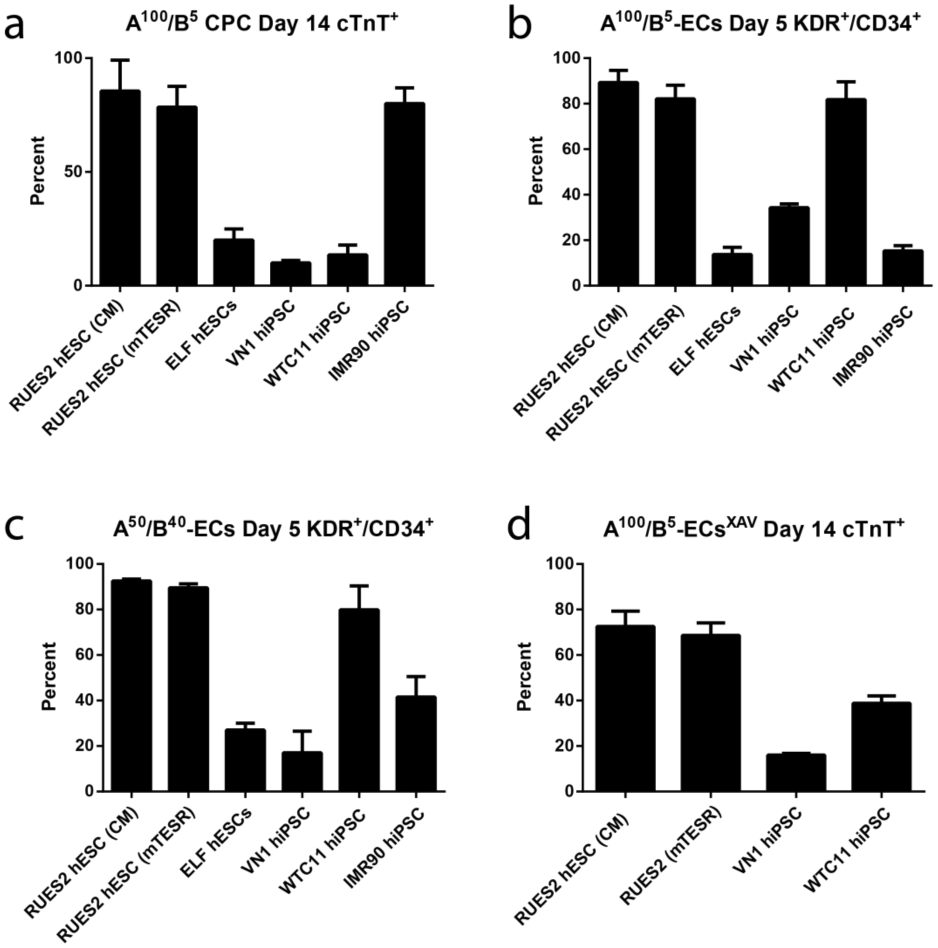

Supplemental Figure 6. Comparison of differentiation potential in multiple human PSC lines. (a) Cardiac differentiation of hPSCs assayed at day 14 for cTnT^{+} cells. (b-c) Endothelial potential was assayed based on differentiation into anterior-derived day 5 cells A^{100}/B^{5}-ECs (b) and posterior-derived day 5 cells A^{50}/B^{40}-ECs (c) based on expression of KDR and CD34. (d) Based on data from (b) RUES2 hESC (CM), RUES2 hESC (mTESR), VN1 hiPSC, and WTC11 hiPSC were chosen to test for efficiency of cell fate conversion from endothelium to cardiomyocytes. n = 4-12 per group.CM: MEF conditioned media. These differentiation efficiencies were compared under a standard protocol optimized for RUES2 cells. These data do not preclude the possibility of increased efficiencies given further protocol optimization.

Development • Supplementary information

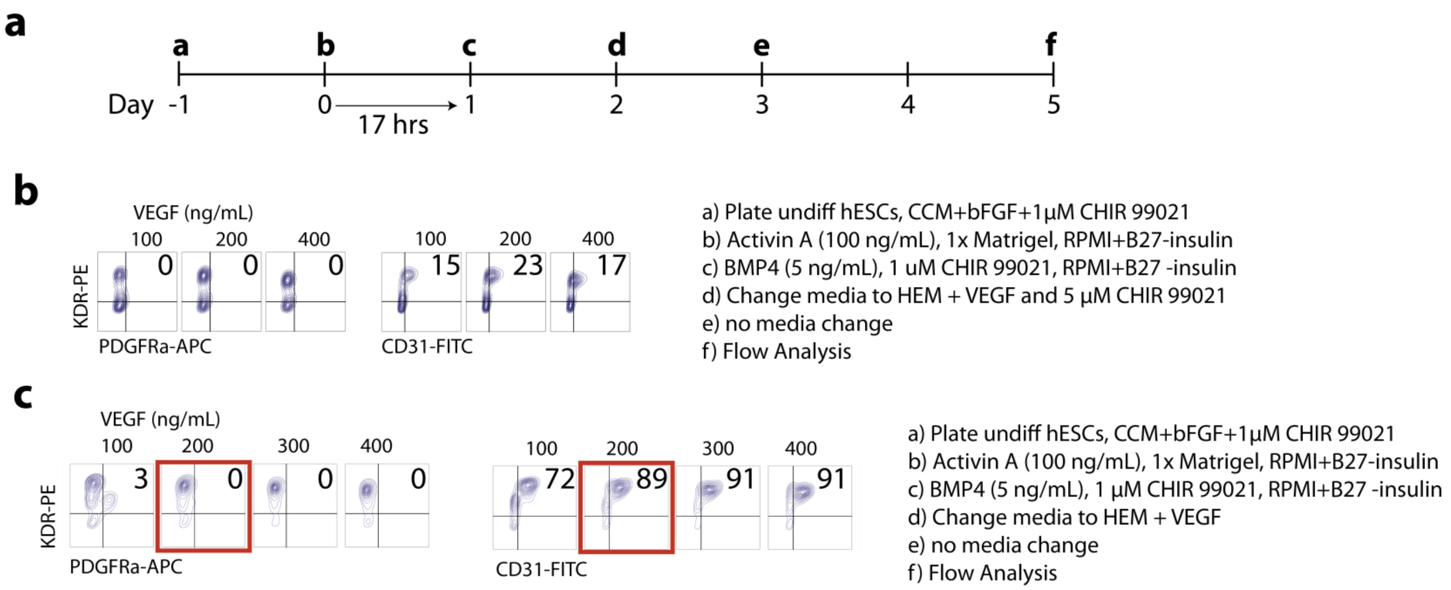

Supplemental Figure 7. Protocol development for endothelial differentiation. (a) Schematic showing time points when variables were manipulated. (b-c) Experiments testing the effect of different doses of VEGF (b-c) in combination with different 5 µM CHIR-99021 (b) or no CHIR-99021 (c) with the final protocol indicated in red boxes in panel c. HEM: Hemogenic endothelial media as described in the methods.

Development • Supplementary information

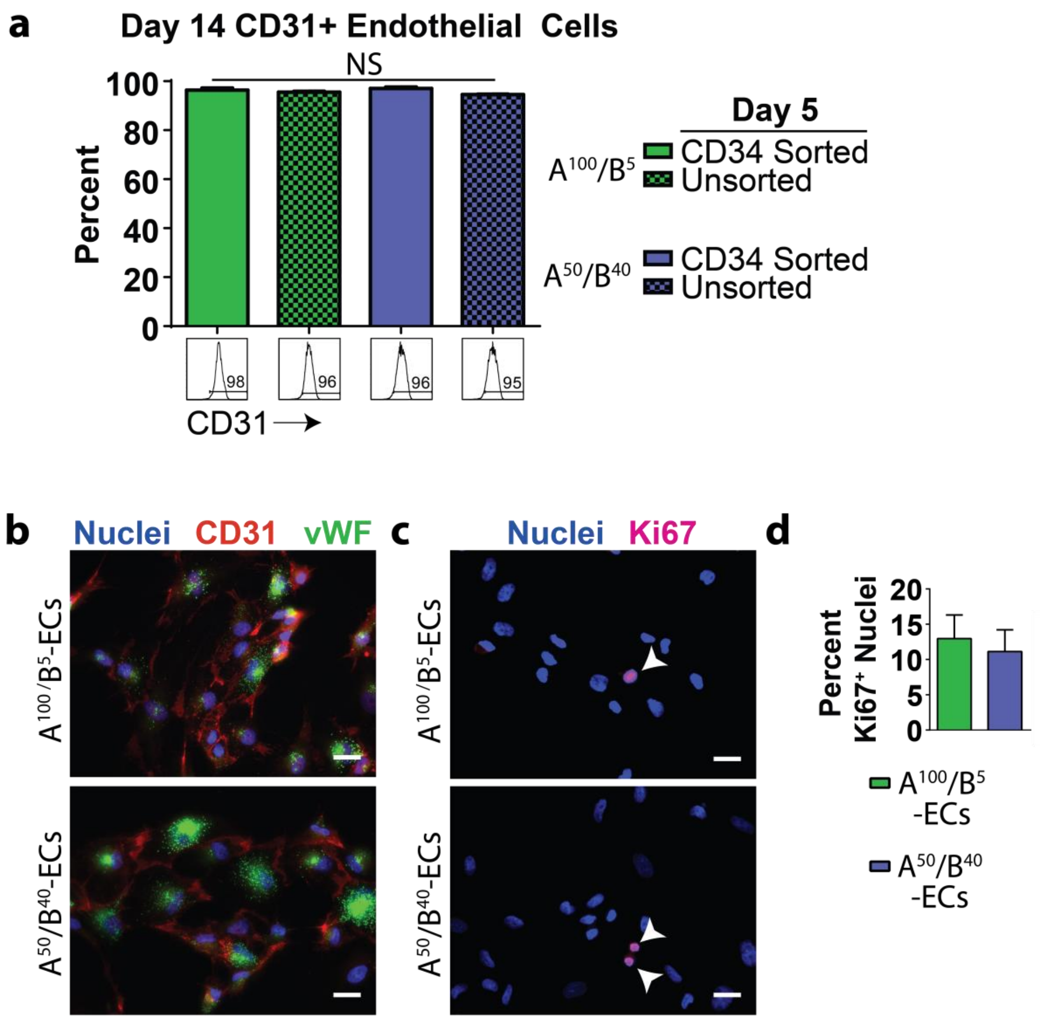

Supplemental Figure 8. Endothelial cell maturation. (a) Flow cytometry analysis of day 14 endothelial cells generated from day 5 CD34 sorted or unsorted A^{100}/B^{5}-$KDR^{+}/CD34^{+}$ cells and A^{50}/B^{40}- $KDR^{+}/CD34^{+}$ cells. (b) IHC analysis of endothelial markers CD31 and vWF in endothelial cells generated from A^{100}/B^{5}-$KDR^{+}/CD34^{+}$ cells and A^{50}/B^{40}- $KDR^{+}/CD34^{+}$ cells. (c and d) Representative images (c) and mean data (d) for cell proliferation based on Ki67 staining in endothelial cells generated from A^{100}/B^{5}- $KDR^{+}/CD34^{+}$ cells and A^{50}/B^{40}- $KDR^{+}/CD34^{+}$ cells. In (b) and (c) scale bar = 20µm.

Development • Supplementary information

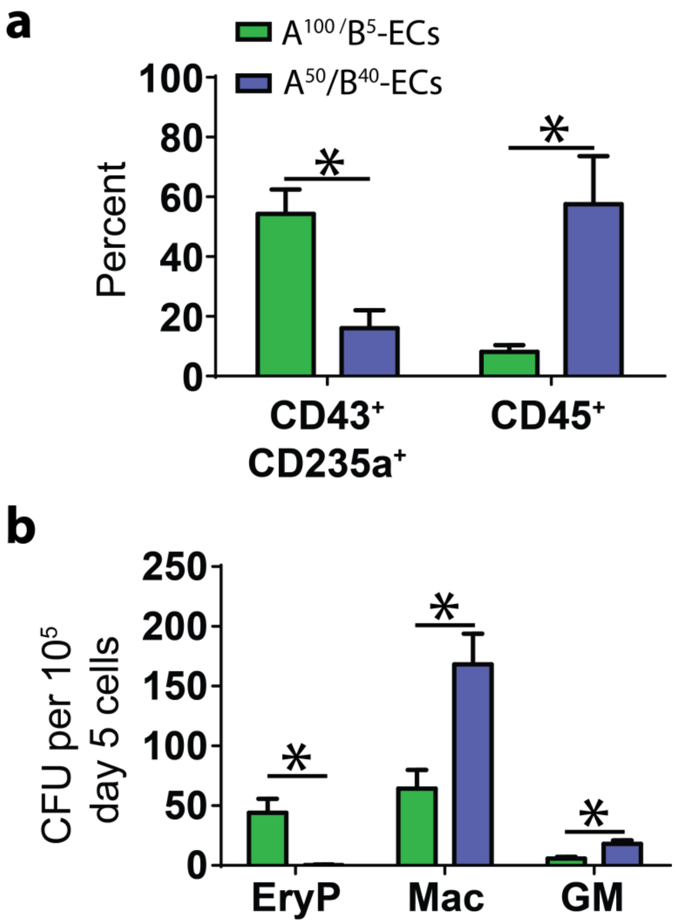

Supplemental Figure 9. Hemogenic potential of day 5 cells cultured for 5 days on OP9 cells. (a) Blood development from A^{100}/B^{5}-ECs and A^{50}/B^{40}-ECs on the basis of CD43/CD235a or CD34/CD45 expression. (b) Colony forming assays for blood types generated from A^{100}/B^{5}-ECs and A^{50}/B^{40}-ECs including primitive erythroid (EryP), macrophage (Mac), and granulocyte/macrophage colonies (GM). n = 3 replicates for each analysis. * $P < 0.05$.

Development • Supplementary information

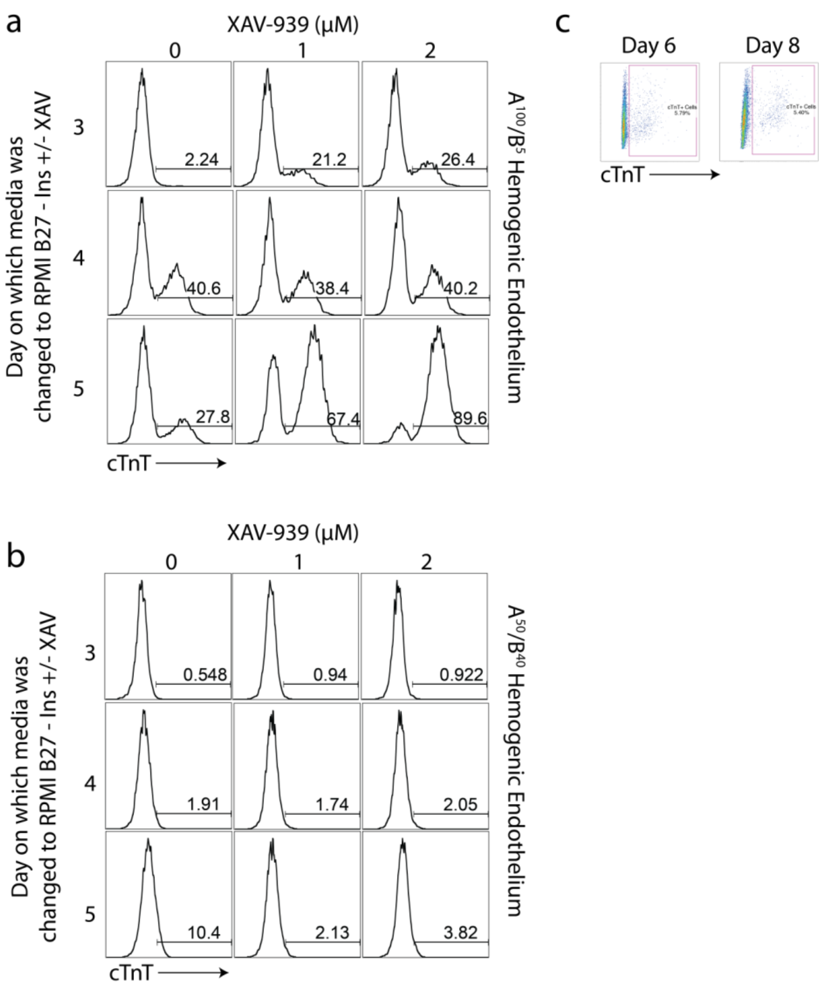

Supplemental Figure 10. Time course analysis of fate conversion from endothelium to cardiomyocytes. (a-b) Both the timing and concentration of XAV-939 treatment were tested for cardiac fate conversion from anterior-derived ECs (A^{100}/B^{5}-ECs) (a) or posterior mesoderm-derived ECs (A^{50}/B^{40}-ECs) (b). (c) Cells were replated and cultured in conditions for endothelial maturation (see methods) then exposed to 2 μM XAV-939 on either day 6 or 8 of differentiation. In all assays, endpoint analysis was performed at day 14 of differentiation by flow cytometry analysis for cardiac troponin T expression.

Development • Supplementary information

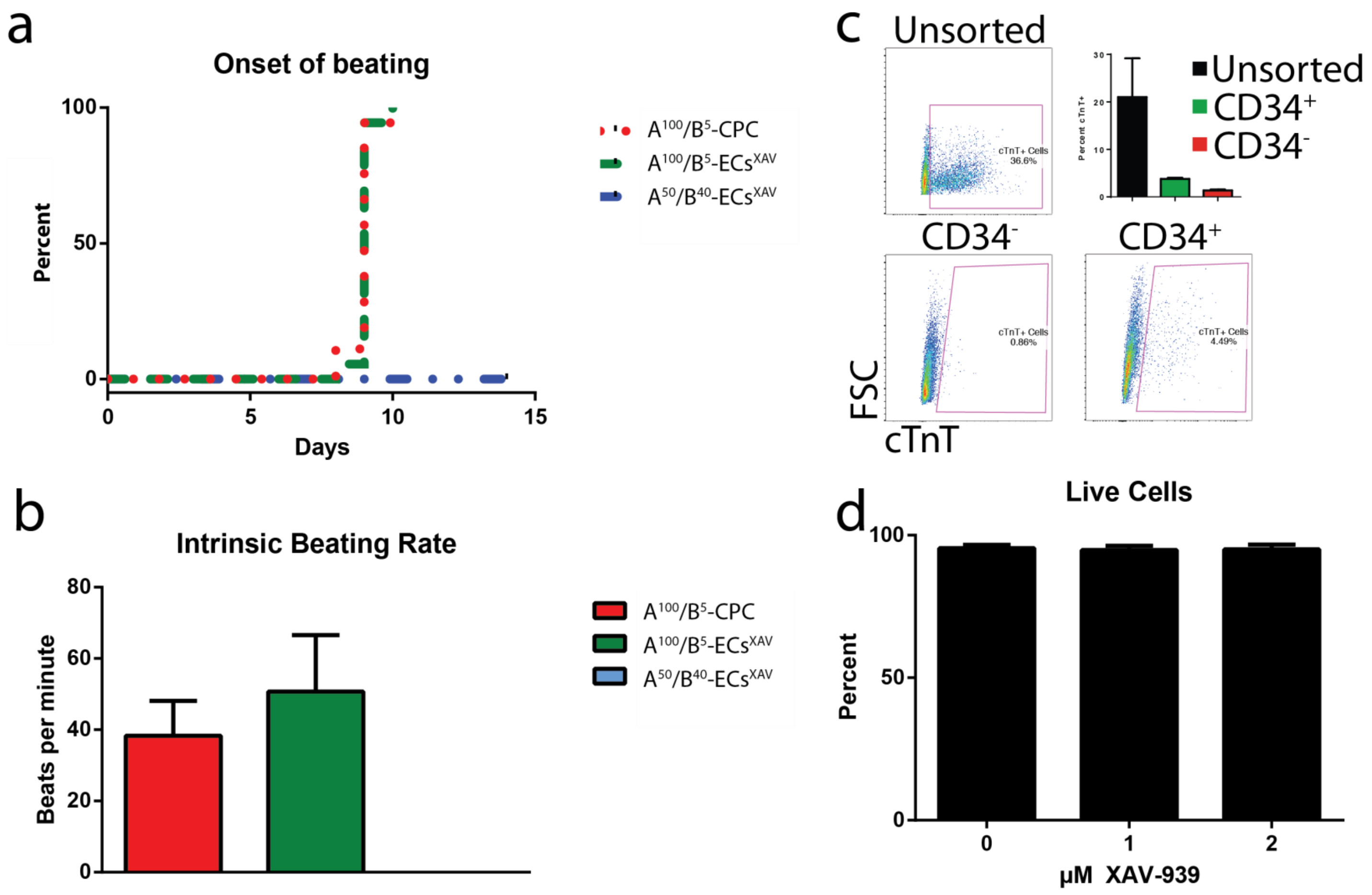

Supplemental Figure 11. Analysis of beating activity in cardiomyocytes generated from different mesodermal origins. (a-b) The onset of beating (a) and the intrinsic beating rate (b) analyzed at day 14 of differentiation was assessed in cells generated by standard cardiac differentiation (A^{100}/B^{5}-CPC) or differentiation by Wnt inhibition from anterior mesoderm-derived ECs (A^{100}/B^{5}-ECs^{XAV}) or cells generated from posterior-mesoderm ECs (A^{50}/B^{40}-ECs^{XAV}). (c) Analysis of cardiac fate switching (A^{100}/B^{5}-ECs^{XAV}) from unsorted control and CD34 positive and negative FACS sorted populations (see methods for modified protocol used in these assays). (d) Live/dead assay at day 7 of differentiation showing percent live cells with increasing doses of XAV-939. n = 6-8 per group.

Development • Supplementary information

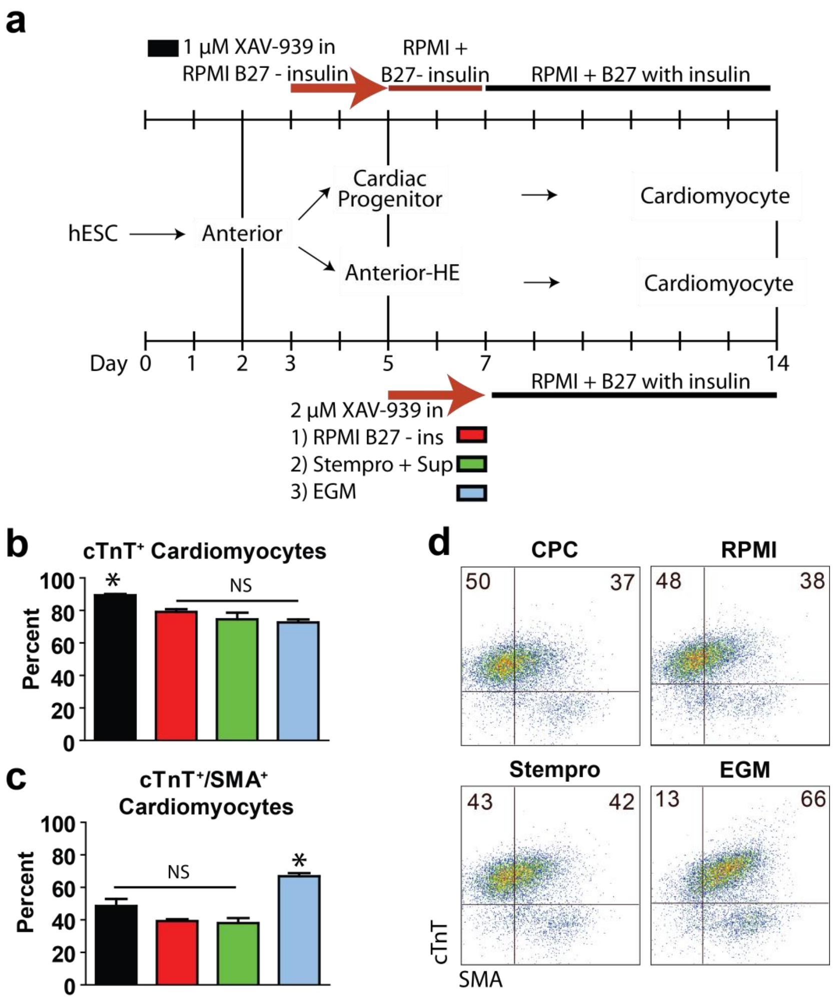

Supplemental Figure 12. Analysis of media effect on endothelial to cardiac cell fate switch. (a) Schematic diagram of experimental approach indicating manipulations of the protocol to test the effect of media on converting HECs to cardiomyocytes. Red arrows indicate the time of XAV-939 addition. Media used included RPMI plus B27 supplement minus insulin (RPMI, red), Stempro34 plus cardiac supplement (Stempro, green) or endothelial growth media (EGM, blue). The black box indicates cardiomyocytes generated using the standard A^{100}/B^{5}-CPC differentiation. (b-d) Cells were collected at day 14 and analyzed by flow cytometry for expression of cardiac troponin T (cTnT) and smooth muscle actin (SMA) showing mean data (b-c) and representative flow plots (d). n = 4 per experiment. * $P < 0.05$.

Development • Supplementary information

Supplemental Table 1. RNA transcripts with significantly increased expression in cardiomyocytes generated from A^{100}/B^{5}-ECs^{XAV} compared to A^{100}/B^{5}-CPCs by RNA-seq.

GENE_NAME	A100/B5-HECSXAV	A100/B5-CPCS	LOG2FOLDCHANGE	PVAL	PADJ
CPA4	45.41972342	0.490976902	-6.531519956	2E-08	2E-06
AFP	332.8730282	4.052093115	-6.36016084	6E-53	5E-49
TTR	32.882012	0.490976902	-6.065499613	1E-09	2E-07
STMN2	22.91050398	0.490976902	-5.54421023	8E-07	7E-05
FGF12	267.2570459	5.954884223	-5.488010714	4E-13	1E-10
SRPX2	17.93659331	0.429860401	-5.38289388	3E-05	0.002
BNC2	16.41435849	0.429860401	-5.254946341	7E-05	0.003
APOA2	17.58860378	0.490976902	-5.162841998	1E-05	7E-04
TMEM71	120.2934697	3.438883212	-5.128474394	5E-25	8E-22
ARSJ	32.08536458	0.920837304	-5.122825287	9E-09	1E-06
TGFBI	734.1386444	23.81953689	-4.945835277	3E-15	1E-12
AC018464.3	25.60106243	0.859720803	-4.896191656	4E-07	4E-05
KRT7	25.19385621	0.920837304	-4.773981871	1E-06	1E-04
COL12A1	553.275552	21.60911839	-4.678286057	1E-61	4E-57
CTHRC1	12.21933024	0.490976902	-4.637366245	9E-04	0.03
SERPINA1	69.11503149	2.823628412	-4.613377361	3E-15	1E-12
ANPEP	11.84173237	0.490976902	-4.592081188	0.001	0.031
DPP4	30.56905143	1.289581204	-4.567097258	7E-08	8E-06
EFEMP1	61.46240571	3.192372312	-4.267003415	8E-10	1E-07
LINC00514	16.42620183	0.920837304	-4.15690884	2E-04	0.007
GREM1	46.18084083	2.640278909	-4.128532197	9E-06	6E-04
NPPB	884.6131132	53.28633061	-4.053209233	4E-61	6E-57
LUM	286.0244278	18.11116357	-3.981187223	7E-28	1E-24
STON2	29.81977736	1.963907609	-3.924470521	2E-07	2E-05
VGLL3	13.37581052	0.920837304	-3.860536227	9E-04	0.03
SPINK1	12.62061478	0.920837304	-3.7766921	0.001	0.034
AC018647.3	176.9892231	12.95283875	-3.772321298	3E-14	1E-11
WISP1	19.09899527	1.411814206	-3.757874597	1E-04	0.005
HOPX	184.9483867	14.05907045	-3.7175496	2E-26	4E-23
COL6A3	256.8887861	19.64521078	-3.708894362	2E-07	2E-05
HBE1	34.722628	2.70139541	-3.684099412	8E-04	0.027
ITGA11	63.83458297	4.972930419	-3.682170079	2E-12	6E-10
RP11-532N4.2	45.50854845	3.561116213	-3.675736148	2E-05	1E-03
CNTN1	200.277325	16.45283846	-3.605590685	4E-22	4E-19
DIAPH3	107.0420142	9.086140035	-3.558365818	7E-11	2E-08
SULF1	195.9579417	16.63823286	-3.557969924	3E-25	5E-22
GDF15	152.6972154	13.13618825	-3.539055141	2E-08	2E-06
MFAP5	20.60938675	1.780558107	-3.532900154	2E-04	0.008
PAPPA	125.202242	11.17432554	-3.486000736	7E-07	6E-05
ACE2	541.0503001	48.31340019	-3.485267429	6E-43	4E-39

Development • Supplementary information

SCG2	36.28631451	3.314605314	-3.452516537	3E-07	3E-05
APOB	52.01061561	4.850697418	-3.422542021	5E-05	0.002
COL21A1	362.5743722	34.43767924	-3.396217127	9E-35	3E-31
NPPA	3009.614172	294.600686	-3.352745853	5E-28	1E-24
SERPINE1	256.1572771	25.5389785	-3.32625715	1E-08	1E-06
EGFLAM	23.31178853	2.332651509	-3.321017001	4E-05	0.002
PID1	23.31178853	2.39376801	-3.283704449	4E-05	0.002
BDNF	22.53882778	2.332651509	-3.27236979	8E-05	0.004
NPY6R	29.83162069	3.192372312	-3.224141537	2E-04	0.008
DACT2	26.35625817	2.823628412	-3.222523415	2E-05	0.001
SNX18P3	20.24363222	2.210418508	-3.195076717	3E-04	0.012
NNMT	16.79195636	1.841674607	-3.188680233	0.002	0.044
KIAA1199	19.86603434	2.210418508	-3.167912458	3E-04	0.013
MAMDC2	23.66569972	2.640278909	-3.164035335	2E-04	0.007
DLK1	78.62011661	8.778512635	-3.162850077	9E-05	0.004
CREB5	442.5996792	49.60502629	-3.157444189	1E-19	8E-17
MARCH1	28.29754254	3.314605314	-3.093767773	4E-05	0.002
PLD5	27.15290559	3.253488813	-3.061047089	1E-04	0.006
ANKRD29	18.71547573	2.271535009	-3.042492275	7E-04	0.024
ANXA1	165.3770469	20.31953718	-3.024819566	9E-19	6E-16
MAOB	220.1255943	28.42372341	-2.953159439	1E-22	2E-19
CDKN2B	80.95084219	10.49795424	-2.946937876	7E-11	1E-08
SYT9	169.6372135	22.28344479	-2.92840851	6E-19	5E-16
SLC45A2	54.58274068	7.305581928	-2.901373735	3E-05	0.002
B3GNT5	32.10905125	4.481953517	-2.840780446	1E-05	7E-04
GDF6	185.2608462	26.5209323	-2.804354612	2E-18	2E-15
AC010136.2	33.99111894	4.911813918	-2.790830043	2E-05	0.001
LYPD1	127.6217926	18.78344508	-2.764341106	6E-15	3E-12
CAV1	321.2904604	48.1259609	-2.738990894	1E-24	2E-21
HFE2	45.46709677	6.875721527	-2.725239865	7E-07	6E-05
ANO4	44.31653815	6.936838028	-2.675495084	1E-06	1E-04
DCLK2	482.2725312	75.6308919	-2.672801103	1E-28	3E-25
ANKRD18A	133.7284969	21.24241938	-2.654286935	4E-14	1E-11
HSPB6	70.30704179	11.17228065	-2.653745474	2E-09	4E-07
MME	55.34385809	8.963907034	-2.626223628	3E-04	0.011
BAALC	36.68759905	5.954884223	-2.623147129	9E-06	6E-04
SLC6A17	31.71368838	5.156279921	-2.620703185	3E-05	0.002
ITGA1	207.4398412	34.19321324	-2.600911098	5E-18	3E-15
CRYM	25.18793454	4.174326117	-2.593117604	0.002	0.045
CSMD1	98.95257385	16.88269887	-2.551191682	5E-11	1E-08
GJA1	818.7898725	142.4201098	-2.523340388	4E-23	5E-20
CD36	397.8640915	69.98567997	-2.507144035	2E-18	1E-15
NTRK2	79.08061784	13.93683745	-2.504420921	4E-09	6E-07
ERVH48-1	23.67162139	4.174326117	-2.503543307	9E-04	0.03
SYT2	31.3005605	5.586140323	-2.486264772	2E-04	0.008
ZNF469	25.19977788	4.543070017	-2.471671572	8E-04	0.025
CASQ2	102.4930747	18.47786257	-2.471656645	4E-04	0.015

Development • Supplementary information

ATP1A3	1992.029147	359.8497219	-2.468772308	6E-24	7E-21
PDE10A	284.0312373	51.322423	-2.468388415	6E-09	8E-07
MARVELD3	36.28039284	6.568094127	-2.46564337	4E-05	0.002
MYH7	92923.20702	16968.01497	-2.453221148	1E-40	6E-37
B3GALNT1	149.8363175	27.5660475	-2.442423019	7E-11	1E-08
GPR1	35.8968733	6.629210628	-2.43694919	5E-05	0.002
MYL2	556.4680634	103.0054103	-2.433578775	2E-16	1E-13
PBK	104.3751424	19.33758338	-2.432298749	2E-06	2E-04
NT5E	21.40603417	3.990976615	-2.423203806	0.001	0.037
FAM83B	42.40486213	7.918791832	-2.420877453	9E-06	6E-04
SYT13	360.2969417	68.20307697	-2.401277673	2E-21	2E-18
SLC5A1	173.5197823	32.96474853	-2.396104167	1E-14	5E-12
GLDC	26.35625817	5.03404692	-2.388354995	5E-04	0.019
ELOVL7	52.35860514	10.00697734	-2.387420397	1E-06	8E-05
SGMS2	23.6893864	4.543070017	-2.382501385	9E-04	0.029
UCHL1	290.7273306	56.90651843	-2.35300088	9E-19	6E-16
OSBPL10	152.1196698	30.13907522	-2.335499656	1E-10	3E-08
CX3CL1	51.94547726	10.31460474	-2.332309606	4E-06	3E-04
STAT4	63.8108963	12.76948925	-2.321101977	2E-07	2E-05
ZC3HAV1L	73.70542262	14.79655826	-2.316509127	1E-07	1E-05
RP11-43F13.3	36.69352072	7.366698429	-2.316435248	4E-05	0.002
KITLG	66.49553307	13.38269915	-2.312888307	1E-07	1E-05
MUC19	44.3639115	8.963907034	-2.30718698	0.002	0.048
FREM2	1847.12039	375.055922	-2.300100273	2E-08	3E-06
CLU	423.2519738	86.06363985	-2.298041034	3E-21	3E-18
C3orf70	88.21402675	18.17432497	-2.279106294	3E-08	3E-06
PDE5A	181.9394471	37.57097995	-2.275767723	5E-12	1E-09
KANK4	124.5417929	25.9077224	-2.265175858	6E-11	1E-08
RAI14	1222.443676	257.2294147	-2.248640462	3E-28	8E-25
SULF2	300.2501807	63.22810165	-2.247527304	9E-18	6E-15
SOX6	71.86480662	15.16325726	-2.244705736	5E-08	6E-06
TAGLN	1820.64292	384.6003206	-2.243016126	7E-30	2E-26
RP11-256I23.3	24.83994502	5.280557819	-2.233899736	0.001	0.037
SLC6A15	77.99519758	16.94586026	-2.202452418	7E-05	0.003
TMSB4X	78.67341162	17.18828137	-2.194450847	1E-07	1E-05
RARRES2	101.1885527	22.28344479	-2.183001909	8E-09	1E-06
FILIP1L	42.77061666	9.516000436	-2.168192765	7E-05	0.003
IQGAP3	144.8446418	32.23135052	-2.167969772	1E-11	2E-09
GPR22	61.85184692	13.81255955	-2.16283599	3E-06	3E-04
AFAP1L2	181.5736926	40.94261197	-2.148880144	5E-07	5E-05
SCN5A	883.5559123	199.6912823	-2.145550081	6E-09	9E-07
EMB	78.73854998	17.92781407	-2.134870553	8E-08	9E-06
TROAP	106.6111213	24.3105138	-2.132705653	2E-09	3E-07
TM4SF1	109.5786092	25.0480016	-2.129198788	2E-07	2E-05
TXNIP	4412.589618	1016.213606	-2.118421893	8E-06	5E-04
SERPINB1	161.9964311	37.87860735	-2.096506835	1E-11	3E-09

F10	45.84469464	10.74242024	-2.093435722	3E-05	0.002
PRR11	197.9110694	46.40856419	-2.092389338	3E-13	1E-10
LINC00842	48.88324261	11.47990805	-2.090228902	3E-05	0.002
KIF20A	438.6993454	103.0706166	-2.089599446	3E-19	2E-16
PHLDB2	644.3933173	151.8261466	-2.085521268	2E-12	6E-10
DEPDC1	207.5523529	49.53982	-2.066814755	8E-10	1E-07
AQP4	46.24597919	11.23339715	-2.041533651	2E-05	0.001
NSG1	178.0568784	43.27730837	-2.040655489	3E-12	9E-10
TOP2A	1270.223733	311.7401202	-2.026666888	4E-24	6E-21
MDGA2	31.70776671	7.796558831	-2.02392686	7E-04	0.024
MGAT4C	30.19145356	7.42781493	-2.023130442	6E-04	0.022
CDKN1A	4493.386206	1109.869546	-2.017412955	1E-10	2E-08
CCDC3	80.94492052	20.01190978	-2.016081697	7E-07	6E-05
KIF18B	68.41313077	17.06604837	-2.003144211	1E-06	8E-05
DRD1	51.18435985	12.76948925	-2.003002219	4E-05	0.002
BIRC5	176.5464869	44.38149518	-1.992017936	6E-12	2E-09
LRRC2	197.9762077	49.7863309	-1.991505459	1E-12	3E-10
RASSF2	32.12089459	8.104186231	-1.986772841	0.001	0.033
ARHGEF6	366.3503509	92.45247426	-1.986440162	6E-16	3E-13
CKAP2L	103.5252	26.1521884	-1.984978409	3E-08	4E-06
LACC1	318.7301786	80.60586721	-1.983378866	1E-15	6E-13
RND3	247.9626356	62.98363565	-1.977073801	1E-13	4E-11
SLC7A5	371.1880632	94.53248018	-1.973268305	8E-10	1E-07
TBX18	114.6176583	29.40567722	-1.962662705	9E-09	1E-06
KIAA0754	123.432686	31.92576802	-1.950931251	4E-09	6E-07
CTGF	888.1581468	229.7815104	-1.950553873	3E-09	5E-07
SYNPR	34.37463848	8.900745636	-1.949346436	6E-04	0.022
MYLK	41.63782305	10.80558164	-1.946117911	2E-04	0.01
LAMC2	27.89625799	7.244465428	-1.94512047	0.002	0.048
CEBPD	95.42983798	24.8626072	-1.940462832	4E-04	0.017
COL8A1	55.36754477	14.42576946	-1.940392275	4E-05	0.002
LAMB1	5558.777387	1453.883959	-1.934855481	6E-25	9E-22
NEK2	103.5725733	27.13414221	-1.932460812	2E-08	3E-06
CMBL	157.7836378	41.86753906	-1.91404358	4E-10	6E-08
KLF9	82.49676368	21.91470089	-1.912438536	1E-06	8E-05
COBL	197.5571582	52.79535371	-1.903787246	1E-11	3E-09
CCRL2	27.51866012	7.42781493	-1.889400452	0.002	0.049
FBN1	401.3795168	108.4877217	-1.887435223	3E-09	5E-07
PIF1	74.49614837	20.19730418	-1.883003094	3E-06	2E-04
CENPF	648.4416928	175.8208534	-1.882870662	6E-05	0.003
SEBOX	44.72374437	12.15423445	-1.879581953	7E-05	0.004
EPN3	50.05156623	13.62716516	-1.876929744	5E-05	0.003
AURKB	79.49966739	21.67023489	-1.875234033	7E-07	6E-05
CDT1	57.71603535	15.83758366	-1.865619961	1E-05	8E-04
GUCY1A2	80.99229387	22.28753458	-1.861547611	2E-06	2E-04
NHSL1	529.1967244	145.7347151	-1.860459553	6E-17	3E-14
KIF14	122.3413441	33.76744263	-1.857207205	1E-04	0.005

Development • Supplementary information

KREMEN2	48.49972307	13.44381565	-1.851033845	2E-04	0.007
PCSK6	250.1571628	69.49470307	-1.847859838	1E-11	3E-09
NCAPH	59.23827017	16.57507147	-1.837514422	2E-05	1E-03
GAS2L3	80.26670647	22.59311709	-1.828918374	1E-06	1E-04
KCNH7	1113.134686	317.16336	-1.811330145	2E-18	2E-15
ENC1	149.7119625	42.85153777	-1.804770619	1E-08	2E-06
LGALS1	32.46888412	9.330606038	-1.799015109	0.002	0.047
COL3A1	3293.358392	947.1242248	-1.797933951	2E-05	0.001
CUX2	656.2336607	188.8368535	-1.797069248	3E-16	1E-13
CIT	136.7907315	39.65712056	-1.786318647	1E-08	1E-06
CRYAB	2323.376796	673.5862377	-1.786288576	3E-21	3E-18
CDC20	198.2412939	57.51972833	-1.785128742	3E-10	5E-08
TK1	56.91938793	16.57507147	-1.779905057	6E-05	0.003
GRID1	165.0112924	48.06688929	-1.779449411	3E-09	5E-07
CPM	178.1338601	52.11898241	-1.773080952	6E-07	5E-05
CRMP1	586.8312453	172.1354593	-1.76940137	1E-05	9E-04
SLFN13	58.50083943	17.31255927	-1.756638316	4E-04	0.017
CDK1	332.0941458	98.28717038	-1.756517275	3E-13	1E-10
SLC6A4	30.58681644	9.086140035	-1.751170509	0.002	0.047
DPYSL5	70.65503131	20.99590848	-1.750684084	2E-05	0.001
NUDT19	96.30346709	28.73135081	-1.744961915	7E-07	6E-05
NXPH2	84.80380258	25.3535841	-1.741939249	4E-06	3E-04
KIF2C	254.818614	76.1850302	-1.741891216	3E-11	6E-09
CEP55	94.40955607	28.36260691	-1.734942754	6E-07	6E-05
PTGER3	60.74866165	18.29655797	-1.731280358	7E-05	0.003
ENPP1	73.36335476	22.22437319	-1.722916882	1E-05	8E-04
ANKRD1	8296.497772	2521.174831	-1.71840629	5E-21	5E-18
INPP5D	94.74570226	28.79246731	-1.71836908	2E-06	1E-04
GJA5	104.3751424	31.80353502	-1.714519129	8E-04	0.026
ASPM	352.7982793	107.8683771	-1.709571541	1E-07	1E-05
NUDT16P	75.61117698	23.14521049	-1.707885829	3E-05	0.002
FUCA2	387.8629751	119.036568	-1.704142228	2E-13	8E-11
ARNTL2	46.63542039	14.36465296	-1.698902984	2E-04	0.008
CSRP1	686.1955581	211.4258808	-1.698467798	1E-15	7E-13
RP11-114H24.5	150.113247	46.47377048	-1.691562693	5E-08	6E-06
GPRIN2	63.38592508	20.01395468	-1.663156258	2E-04	0.006
NEAT1	737.7575159	233.3569409	-1.660608334	6E-15	3E-12
RASD2	61.84592525	19.64316588	-1.654651079	9E-04	0.03
CCNB2	222.060957	70.65796148	-1.652031727	8E-10	1E-07
CENPA	39.7498337	12.70837275	-1.645169513	9E-04	0.03
ATP8A1	784.6712547	250.9057866	-1.644942606	4E-10	7E-08
FRMPD4	128.41844	41.06893476	-1.644732946	9E-08	1E-05
RP11-115D19.1	58.08178988	18.60214047	-1.642617277	1E-04	0.006
PVT1	45.46709677	14.60911896	-1.637953711	5E-04	0.017
NDC80	92.50380171	29.83553762	-1.632480885	2E-06	2E-04
CENPE	242.7058738	78.5849329	-1.626884388	3E-07	3E-05

Development • Supplementary information

CASC5	125.7456466	40.88763016	-1.620772102	2E-07	2E-05
ASF1B	46.59989038	15.16325726	-1.619746865	8E-04	0.025
S100A10	493.624154	160.6473716	-1.619515613	2E-13	8E-11
LRP2	193.2318532	62.92251915	-1.618684598	2E-08	2E-06
FOXM1	185.4384963	60.40447324	-1.618213476	7E-04	0.023
FGF1	272.9091707	88.95247455	-1.61731423	3E-08	3E-06
CCND1	653.8910918	213.1453224	-1.617212974	1E-13	5E-11
ST6GAL2	48.91877262	15.96186156	-1.61575929	4E-04	0.014
SNCAIP	217.4409575	71.02670538	-1.614190243	8E-10	1E-07
HAND1	1557.688881	509.3145885	-1.612778165	3E-09	5E-07
MKI67	554.1965545	181.3479221	-1.611637527	3E-10	5E-08
LYPD6B	81.03374555	26.51888741	-1.611502578	7E-06	4E-04
ABCC9	262.1824669	85.88233524	-1.610137885	8E-11	2E-08
DLGAP5	191.1498377	62.68009804	-1.608624634	4E-05	0.002
CYBRD1	63.46290677	20.81255898	-1.608459241	2E-04	0.008
TNS3	522.9064484	172.0070916	-1.604084815	7E-13	2E-10
ARHGAP11A	169.7141952	55.92660952	-1.601500463	2E-07	2E-05
NCAPG	107.4432987	35.42372284	-1.60078776	0.001	0.034
TNFRSF10C	110.0272671	36.34456014	-1.598049749	2E-06	2E-04
ASPG	58.84882896	19.46186127	-1.596364015	2E-04	0.007
PTTG1	393.9756011	130.5062515	-1.59398737	5E-12	1E-09
ADAMTS6	544.8618088	180.7937838	-1.591545296	3E-13	9E-11
CDCA8	133.4278807	44.63209587	-1.579906699	2E-04	0.007
GYG1	1791.217117	600.1455458	-1.577555893	5E-16	3E-13
GTSE1	139.0740837	46.65507509	-1.575747671	2E-07	2E-05
MATN3	97.10011451	32.66121093	-1.571894717	5E-05	0.003
SPARC	6185.548326	2082.33304	-1.570700664	5E-17	3E-14
MYO7A	56.52402505	19.02995598	-1.570591979	4E-04	0.014
ABCA8	265.6578294	89.56977425	-1.568485357	4E-07	3E-05
TNS1	1588.072972	536.0006966	-1.566970425	1E-15	6E-13
ARRDC4	543.2639813	183.621502	-1.564918391	1E-04	0.005
TCEAL7	62.2472098	21.11814148	-1.559526296	3E-04	0.011
SLC16A14	44.69413602	15.16325726	-1.559505863	0.001	0.037
PARM1	1967.349087	669.3876032	-1.555339234	2E-15	9E-13
LRP8	227.3947005	77.47256651	-1.553441193	1E-09	2E-07
RRM2	61.17363287	20.87367548	-1.551225299	7E-04	0.022
TLN2	2009.463421	687.3826685	-1.547624934	6E-14	2E-11
UBE2C	177.277996	60.83228875	-1.543104287	3E-08	4E-06
LY6E	130.2472127	44.81340048	-1.53925039	1E-06	1E-04
CCNA2	353.6719084	121.797035	-1.53793262	6E-04	0.022
NRCAM	152.4498943	52.5508877	-1.53654811	2E-07	2E-05
ANLN	592.9557146	204.7416884	-1.534119474	1E-04	0.005
SEMA5B	43.18374454	14.91674636	-1.533555458	0.001	0.032
FAM64A	40.12743157	13.93479256	-1.525897291	0.002	0.045
VAT1L	84.80972425	29.46883861	-1.525039489	4E-05	0.002
FAM83D	110.8357579	38.55497865	-1.523434326	2E-06	2E-04
NUSAP1	302.3335851	105.2851249	-1.52183963	1E-08	2E-06

Development • Supplementary information

SULT4A1	154.2431369	54.02381841	-1.513538779	1E-04	0.005
SYNPO	334.6070542	117.2457854	-1.512931797	3E-10	6E-08
ME1	257.9519086	90.48652176	-1.511327302	1E-09	2E-07
RAB6B	862.2578571	302.6905579	-1.510275697	3E-12	9E-10
ITPR2	89.05212586	31.31051322	-1.508002896	2E-05	1E-03
ATRNL1	337.6633672	118.7350753	-1.50783949	9E-10	2E-07
TNNT3	79.45229404	28.17516762	-1.495664682	6E-05	0.003
CENPN	86.37341075	30.69321352	-1.492667559	2E-05	0.001
CDC6	46.64726373	16.63618797	-1.487467566	0.001	0.039
KIAA1244	244.900401	87.54066035	-1.484169964	7E-09	1E-06
NUF2	87.93709725	31.67721222	-1.473026535	6E-04	0.02
SYTL5	1007.66959	363.1277495	-1.472473577	1E-13	5E-11
GYPC	524.7648294	189.1892383	-1.471841006	1E-07	2E-05
HMMR	181.1605647	65.44056506	-1.469011831	5E-08	6E-06
MYBL2	95.53050634	34.62307364	-1.464227707	2E-05	1E-03
EHD2	119.5738039	43.39954138	-1.462149658	4E-06	3E-04
ALS2CL	479.451696	174.3458778	-1.45943322	6E-11	1E-08
KIF3C	199.043863	72.68503049	-1.453356214	9E-08	1E-05
SLC16A7	94.78123227	34.81255783	-1.444993599	3E-04	0.011
RP1-46F2.2	863.8852931	317.3017218	-1.444984414	3E-07	3E-05
GPR39	54.27620283	20.01395468	-1.439313527	6E-04	0.021
PIK3R3	192.949002	71.15302818	-1.43922252	1E-07	2E-05
EDIL3	267.4806804	98.78223708	-1.437111146	9E-09	1E-06
ARAP2	90.57436068	33.45981523	-1.436673239	3E-05	0.002
PLEKHG1	85.582685	31.61609572	-1.436659722	6E-05	0.003
CSRP2	1700.826328	630.1542084	-1.432459008	9E-14	3E-11
ACOT7	142.9329658	53.16000781	-1.426925474	7E-07	6E-05
SDSL	68.75519862	25.600095	-1.425319637	4E-04	0.014
STARD4	3224.232174	1204.826212	-1.420130572	2E-08	3E-06
TMEM88	198.2945889	74.16000609	-1.418932042	2E-07	2E-05
CDCA3	90.94603688	34.19525813	-1.411214491	5E-05	0.002
TACC3	186.8778278	70.29126248	-1.410678138	1E-07	2E-05
HTATIP2	80.21933312	30.20428152	-1.409196904	2E-04	0.007
IRF6	281.605765	106.1957377	-1.406951006	6E-08	6E-06
CALD1	1447.731285	547.3788206	-1.403182328	6E-13	2E-10
LZTS1	113.8743059	43.34251467	-1.393587496	1E-05	8E-04
TTK	103.614025	39.47377105	-1.392253041	0.001	0.032
CAV2	575.0473407	219.1695027	-1.391633668	9E-11	2E-08
COL5A2	1430.311632	545.8345489	-1.389793893	6E-12	1E-09
CDC25C	52.75396802	20.13618768	-1.389489045	8E-04	0.026
AC108142.1	126.0817928	48.25023879	-1.385751966	6E-06	4E-04
KCNK3	113.8210108	43.58084598	-1.38500079	0.001	0.032
AHNAK	10035.5914	3852.77579	-1.3811555	3E-14	1E-11
LAMA2	5246.90421	2016.336062	-1.37973034	2E-11	4E-09
RP11-471J12.1	3185.63151	1226.958796	-1.376492596	5E-14	2E-11
MBNL3	47.0248616	18.17228007	-1.371684252	0.002	0.044

Development • Supplementary information

ADCY6	5309.185927	2059.794905	-1.365989971	1E-13	5E-11
KIF4A	105.1244165	40.88354036	-1.362505754	2E-04	0.007
DTL	70.32480679	27.38065311	-1.360876825	3E-04	0.011
PDLIM1	2587.399879	1008.919833	-1.358691493	3E-13	9E-11
HSPB2	91.28810474	35.60502744	-1.358345918	1E-04	0.005
INPP4B	342.0242021	133.8351711	-1.353641118	5E-09	8E-07
ABCB4	113.9216792	44.75228398	-1.348009098	2E-04	0.007
RNF150	1021.298644	402.2470909	-1.344250902	2E-11	5E-09
SHROOM3	807.5862914	320.5715698	-1.332969937	9E-11	2E-08
EPS8	195.309336	77.53368301	-1.33286581	3E-07	3E-05
COL9A1	157.8961495	62.80028615	-1.330132952	7E-04	0.024
ADAM19	1240.338818	494.3898929	-1.327013114	1E-11	2E-09
SLC1A3	1312.185859	523.6915108	-1.325182956	1E-11	3E-09
GAS7	1269.550052	506.8374405	-1.324722268	1E-11	3E-09
SLC27A6	1150.424906	460.0662671	-1.32225323	2E-11	5E-09
CNKSR2	115.408384	46.35153748	-1.316058936	3E-05	0.002
CFLAR	180.0218494	72.38762757	-1.314356979	0.002	0.047
PDLIM3	152.7505104	61.63702774	-1.309308002	2E-05	0.001
BUB1B	238.1214043	96.13786838	-1.308520587	3E-07	3E-05
SSPN	124.9371558	50.5849352	-1.30442289	2E-05	0.001
ANXA3	896.2977383	364.2726039	-1.298959559	5E-06	3E-04
SPECC1	779.0118194	316.6417097	-1.29879391	2E-10	4E-08
ADAMTS19	1809.796028	737.859685	-1.294408711	3E-04	0.013
TES	476.7670593	194.4820655	-1.29364744	5E-09	7E-07
ACOT2	158.6158152	64.76828355	-1.29217721	3E-06	2E-04
CDS1	75.30463913	30.75637492	-1.291853274	3E-04	0.01
PCDH20	426.9405164	174.535362	-1.290515715	8E-07	7E-05
HSPA4L	213.2755376	87.24121253	-1.289636774	3E-05	0.002
UCP2	325.8571648	133.8229017	-1.283914684	5E-08	6E-06
EIF5A2	204.0177737	83.98363393	-1.280514723	2E-06	1E-04
CHST15	114.1986087	47.02790878	-1.279955989	1E-04	0.005
CYP2J2	640.8528164	264.5125028	-1.276657137	3E-07	3E-05
MELK	66.89681762	27.62511911	-1.275956896	5E-04	0.018
COL1A1	6816.585718	2820.722555	-1.272984535	2E-05	1E-03
AKAP6	1045.626181	433.4292365	-1.270498793	3E-09	4E-07
PKMYT1	96.98760281	40.33349186	-1.265822038	4E-04	0.015
CSDC2	106.9117375	44.50781797	-1.264289572	3E-04	0.01
STK39	3765.854098	1582.601473	-1.250679101	2E-10	3E-08
SERPINB9	86.01357789	36.15712084	-1.250284616	5E-04	0.018
LOX	908.8162958	383.2882456	-1.245558949	1E-06	9E-05
ATF3	367.3587895	155.0021597	-1.244901476	2E-05	0.001
RIMS4	260.1997308	109.82615	-1.244397863	4E-07	4E-05
PLK1	282.4083341	119.6436432	-1.239038918	1E-07	1E-05
PRSS35	1786.700564	757.9715643	-1.23708224	6E-06	4E-04
MMP2	858.3219934	364.5252494	-1.235500217	2E-09	3E-07
C3orf52	116.588551	49.72725929	-1.229317298	7E-05	0.003
PHF17	311.395934	133.7679199	-1.219017935	2E-07	2E-05

Development • Supplementary information

COL4A1	10670.21001	4590.608431	-1.216831288	7E-12	2E-09
AKR1B1	1034.744125	445.2574395	-1.216562436	2E-09	3E-07
SPOCK1	500.2624195	215.5838478	-1.214435988	1E-04	0.005
SYNJ2	119.6152556	51.566889	-1.213884485	6E-05	0.003
COL14A1	287.6532526	124.241695	-1.211181366	5E-05	0.003
GADD45G	72.18910947	31.24735182	-1.208047279	0.002	0.047
ALCAM	1394.700388	604.5909215	-1.205924012	4E-10	6E-08
THNSL2	184.4345905	80.11489031	-1.202966942	3E-05	0.002
TMTC1	458.3403564	199.2716464	-1.20168287	1E-07	1E-05
MICAL3	2667.530387	1163.378309	-1.197184395	2E-10	3E-08
RP11-6F2.6	1018.313391	445.8604249	-1.191517571	8E-10	1E-07
TMSB15B	105.8559256	46.35153748	-1.191412931	2E-04	0.008
CCNB1	467.8468304	204.8618765	-1.191384747	6E-06	4E-04
CPNE8	76.44335441	33.58204823	-1.186700861	8E-04	0.026
A4GALT	291.7535342	128.1817796	-1.186558928	3E-06	2E-04
CEP152	127.5921842	56.29944321	-1.180347399	2E-04	0.007
MLLT11	1666.867595	735.8794182	-1.179598221	3E-06	2E-04
DOK7	244.8234193	108.1087533	-1.17925823	6E-06	4E-04
KIAA1522	630.1602537	278.7040307	-1.176984894	5E-08	6E-06
ADAMTSL1	443.3371099	196.8717437	-1.171148069	4E-07	4E-05
STRN	2749.804906	1222.252825	-1.169786525	2E-10	4E-08
GALE	272.3953744	121.1186188	-1.169281547	1E-06	1E-04
EPAS1	121.5269316	54.39051742	-1.15984901	9E-05	0.004
MYRF	2506.428494	1123.658027	-1.157430042	2E-09	3E-07
OPTN	883.9645075	396.8665636	-1.155334425	9E-09	1E-06
CDCA5	77.95966756	35.05293404	-1.153192731	9E-04	0.03
NPNT	1696.096595	762.673445	-1.153080962	4E-08	4E-06
CACNA2D2	409.3919754	185.8848574	-1.139073574	1E-06	9E-05
SLC16A12	241.0355972	109.5919085	-1.137104942	2E-04	0.009
FAM65B	244.5287248	111.1809375	-1.137094496	6E-06	4E-04
INHBA	344.1950427	156.605503	-1.13609141	1E-06	1E-04
SGK1	81.38173507	37.08204794	-1.133984117	0.002	0.044
RAP1GAP	641.9901428	293.1786473	-1.130771113	1E-04	0.005
TFRC	3594.762932	1644.241006	-1.128474851	1E-09	2E-07
MURC	247.6916278	114.0636375	-1.118706158	3E-05	0.002
GOLT1B	410.4077246	189.8881034	-1.111908362	5E-07	5E-05
PRC1	443.0187287	205.0390913	-1.1114687	7E-05	0.003
SPAG5	74.13039384	34.31544624	-1.111207061	0.002	0.049
ARHGAP23	559.2401363	259.9226305	-1.105385656	3E-07	3E-05
DENND3	441.7260501	205.3548983	-1.105032555	4E-07	4E-05
SLITRK4	199.8286671	93.01070236	-1.103294929	3E-05	0.002
KPNA2	1058.636237	496.2111186	-1.093180977	3E-08	3E-06
MROH1	190.6478848	89.51274754	-1.090745468	5E-05	0.003
GPRIN3	1334.264186	629.9669995	-1.082696189	1E-06	9E-05
SYNDIG1	153.9306774	72.80317369	-1.080207532	1E-04	0.006
HEY2	1806.233596	854.3148152	-1.080144778	1E-07	1E-05
C12orf5	121.8689995	57.71125742	-1.078406516	6E-04	0.02

Development • Supplementary information

TPX2	474.2719159	224.9939744	-1.075828077	3E-07	3E-05
TBC1D9	208.6081648	99.70102948	-1.065115318	4E-05	0.002
TMEM176A	172.5869364	82.50661343	-1.064741597	5E-04	0.019
FMOD	136.7907315	65.43852016	-1.063758453	2E-04	0.008
SRPX	572.0384011	273.6679389	-1.063685571	2E-07	2E-05
STARD9	558.0777343	267.1121141	-1.063020681	1E-06	8E-05
EDA2R	307.1772191	147.1608436	-1.061677375	9E-06	6E-04
TCTEX1D1	132.9496145	63.78837464	-1.05951417	4E-04	0.017
TUBA1C	192.5358742	92.57879706	-1.056373558	9E-05	0.004
CTNND2	276.2957082	132.9693156	-1.055119788	6E-06	4E-04
LGALS3	166.2565977	80.04763912	-1.054480835	3E-04	0.013
LIMCH1	221.6241424	107.0004767	-1.050497824	2E-05	0.001
JUN	1558.947785	752.6641924	-1.050494365	5E-05	0.003
ATP2B4	11323.40059	5481.808091	-1.046583559	3E-09	5E-07
RAB31	699.0516508	339.0596568	-1.043859921	6E-07	6E-05
ICA1	408.8262731	198.719553	-1.04075408	2E-06	2E-04
STRIP2	1476.165026	720.4494315	-1.034884939	2E-07	2E-05
RP4-575N6.2	107.384082	52.42456491	-1.034465265	6E-04	0.022
WIPF3	418.7918594	205.0268219	-1.030420741	2E-04	0.009
EHD3	1302.396534	638.3744929	-1.028693852	8E-08	9E-06
PAQR8	576.5162805	282.8274648	-1.027439125	1E-06	9E-05
MYBPC3	26459.04411	12992.16829	-1.026118717	8E-09	1E-06
BCL11A	209.3692822	102.8261506	-1.025842576	6E-05	0.003
PTPN14	174.9591137	86.06772964	-1.023473499	2E-04	0.008
NCAPG2	158.173079	77.96149851	-1.020670348	2E-04	0.008
ZNF697	488.3318621	241.3348043	-1.016825922	2E-06	2E-04
OLFML3	293.8073302	145.2437382	-1.016394424	1E-05	9E-04
TNFRSF12A	825.1438979	409.2123271	-1.011796129	0.001	0.038
C12orf75	1420.171174	704.7604341	-1.010859991	1E-07	1E-05
NDNF	415.7473898	206.3816094	-1.010392787	8E-06	5E-04
KCNJ8	388.169513	192.8746324	-1.009023405	2E-05	0.001
GCNT2	1112.94066	553.1971575	-1.008511023	3E-07	3E-05
PGF	172.9941427	86.01070293	-1.00813509	5E-04	0.018
STAMBPL1	150.131012	74.6468932	-1.008067898	4E-04	0.014
KIF11	219.4059286	109.092752	-1.008047255	2E-04	0.007
AGPAT9	604.117844	300.732785	-1.006347846	5E-06	4E-04
GPC1	1617.005888	807.6985932	-1.001436001	4E-07	4E-05

Development • Supplementary information

Supplemental Table 2. RNA transcripts with significantly increased expression in cardiomyocytes generated from A^{100}/B^{5}-CPCs compared to A^{100}/B^{5}-ECs^{XAV} by RNA-seq.

GENE_NAME	A100/B5-HECSXAV	A100/B5-CPCS	LOG2FOLDCHANGE	PVAL	PADJ
UNCX	2.678715106	172.3819702	6.007923926	2E-34	8E-31
FAM5B	3.823352054	82.51274812	4.431707076	3E-12	7E-10
WSCD2	4.973910672	103.8774005	4.384357408	3E-13	9E-11
SMOC1	7.646704109	126.8413064	4.052042781	5E-07	5E-05
PPFIA2	27.51866012	414.8455001	3.914092005	2E-06	0.0001
TBX21	6.112625952	91.16698285	3.898647238	7E-16	3E-13
RP11-834C11.4	1.522234819	21.79451278	3.839702122	1E-05	0.0007
SPOCK3	15.27564321	195.2929391	3.676334755	6E-11	1E-08
ASTN1	38.62888342	488.9505243	3.661936608	2E-05	0.0009
CSNK1A1P1	1.144636949	14.42781435	3.655890777	1E-03	0.0306
NPAS4	3.805587048	40.39869815	3.408117877	5E-04	0.0169
SIX1	2.678715106	27.31953661	3.350319956	4E-05	0.0019
GRIA4	16.41435849	164.4754477	3.324841967	5E-07	4E-05
STAC3	50.0397229	493.287751	3.301283758	1E-22	1E-19
AC003090.1	1.917597696	18.72437347	3.28754546	0.001	0.0397
C3orf80	7.64078244	70.41349548	3.204059675	1E-10	2E-08
TNMD	3.835195392	34.37860763	3.16413913	2E-05	0.0014
JAKMIP2	24.01368925	215.1214994	3.163222747	9E-12	2E-09
LRP1B	53.88676163	456.9025233	3.083883616	8E-05	0.0037
CX3CR1	4.973910672	39.47581595	2.988516583	4E-06	0.0003
ZNF385D	27.12329725	213.9500614	2.979669618	5E-21	5E-18
TNNC2	22.17307325	173.7958293	2.97051281	1E-17	6E-15
DCP1B	36.28039284	282.8783568	2.962919817	5E-23	6E-20
ANO3	2.289273898	17.68130317	2.949262622	9E-04	0.0297
TMEM255A	8.018380311	60.28633003	2.910448156	6E-09	9E-07
CCL21	2.678715106	19.82856028	2.887966873	0.002	0.0442
WNT2	203.5158207	1457.789741	2.840569798	6E-09	8E-07
ARTN	9.959664681	70.29535227	2.819260229	4E-06	0.0003
DGKB	6.885586699	47.02790878	2.771865688	2E-05	0.0013
HOXB4	22.91050398	151.6325726	2.726498596	1E-15	6E-13
PIANP	9.941899675	65.56279806	2.721283976	4E-08	5E-06
MAOA	54.57681901	346.8155481	2.667808362	2E-06	0.0002
RSPO3	1057.681094	6269.265314	2.567391686	2E-16	1E-13
BMP2	902.3734454	5080.156266	2.493076357	7E-37	3E-33
HAS2	531.1543849	2931.875555	2.464620709	6E-06	0.0004
RGMA	6.100782615	33.64316473	2.463247193	2E-05	0.0013
RP3-340I3.1	5.36335188	28.05497951	2.387050045	0.002	0.0461
LRRN4CL	4.967989003	25.2313511	2.344483577	0.001	0.0353
MYOM2	91.63609426	462.7331297	2.336192519	3E-04	0.0107
CACNA2D4	5.351508542	27.01190921	2.335578077	9E-04	0.0288
LRRTM3	12.60877144	61.39256174	2.28363616	1E-06	9E-05

Development • Supplementary information

LIPH	11.08061496	51.5098623	2.216810732	2E-04	0.0065
ITGA10	19.86603434	92.15302645	2.213727636	2E-04	0.0065
LMO2	82.55598037	380.0879241	2.202888556	4E-06	0.0003
GJD2	15.26972154	68.45163277	2.164411201	3E-07	3E-05
HS3ST3B1	13.75340839	61.32940034	2.156789651	3E-06	0.0002
PLEKHF1	6.879665031	29.65014322	2.107628846	0.001	0.0388
EML1	244.1392836	1034.379521	2.08298926	2E-22	2E-19
KIAA0319	68.0355329	288.1752738	2.08258623	2E-13	6E-11
MYH11	9.918213001	41.98977207	2.081885843	4E-05	0.002
NELL1	19.11676027	80.79330651	2.079397725	7E-07	6E-05
PPP1R3A	179.1245336	748.0888648	2.062246709	1E-04	0.0045
IRX1	35.86726495	149.3569478	2.058024708	2E-08	2E-06
BST2	69.11503149	282.0370401	2.028813231	5E-12	1E-09
IGLON5	25.57145408	102.7100523	2.005971281	8E-09	1E-06
SNCA	180.3047006	723.3830235	2.004322732	2E-17	1E-14
KIAA1024	45.45525343	181.1604828	1.994749343	3E-11	6E-09
CNRIP1	15.65916275	62.13004954	1.988284127	6E-06	0.0004
IFITM1	38.15061718	148.6887561	1.962517253	4E-11	9E-09
KCNIP2	1534.275036	5892.285921	1.941270313	3E-24	4E-21
SCD5	49.65028169	189.8819687	1.935229105	3E-11	6E-09
BMPER	337.3331426	1289.435326	1.93449344	2E-08	2E-06
RP11-456K23.1	10.31357588	38.85851625	1.913686191	5E-04	0.0169
FER1L6	19.13452528	71.70716648	1.905939202	3E-04	0.0109
C5orf38	24.02553259	87.66902804	1.867498841	7E-06	0.0004
FOXC1	15.26972154	54.02586331	1.822976467	4E-05	0.002
KIRREL2	15.30525156	53.71823591	1.811385171	3E-04	0.0132
TSPAN15	313.8969991	1065.802043	1.763576362	3E-18	2E-15
RYR1	264.3947591	895.2842413	1.759652213	8E-12	2E-09
LRRN1	25.96681696	87.11693463	1.746284013	2E-06	0.0002
KCNMA1	15.64139774	51.6300504	1.722841566	6E-05	0.003
DACT1	1179.20978	3867.048423	1.713412435	3E-20	3E-17
KBTBD13	20.23771055	66.17805286	1.709306754	3E-05	0.0017
GPR183	28.65145373	92.69898516	1.693945204	4E-06	0.0003
IRX2	126.7422418	402.4222609	1.666812674	2E-13	6E-11
AC159540.1	18.35564286	58.13498313	1.663182934	4E-04	0.0164
NCEH1	133.3390557	416.0555607	1.641676786	3E-11	6E-09
GPR61	17.96027999	55.98977092	1.640353437	3E-04	0.0113
VAMP8	63.76944462	198.5362035	1.638464888	2E-09	4E-07
HOXB2	97.70134687	303.5177908	1.635330727	4E-12	1E-09
EPHB2	334.5833675	1028.338982	1.619878279	2E-15	9E-13
EPHA8	21.37642583	65.62391456	1.618201003	5E-05	0.0025
SLC24A3	60.75458332	186.381969	1.617197143	3E-08	3E-06
ADRA1B	33.23000153	101.8462417	1.615834479	3E-06	0.0002
RGL3	118.0515691	361.4185324	1.614253267	2E-08	3E-06
NELL2	35.5429621	108.7240081	1.613034724	4E-06	0.0003
DERL3	16.78011302	51.3244679	1.612894332	1E-04	0.0062
PHACTR3	101.2418477	301.9612497	1.576557679	8E-04	0.0263

MYOZ1	131.0497818	389.9522194	1.573182411	5E-11	1E-08
RP11-867G23.10	35.87910829	105.649779	1.558073809	2E-06	0.0001
SEPT4	32.48664913	95.64689147	1.557871139	2E-05	0.0013
ABRA	666.7485734	1950.304781	1.548484858	1E-08	2E-06
AQP3	16.40843682	47.94670119	1.546993754	4E-04	0.0144
PITX3	23.30586686	68.08288886	1.546599079	2E-04	0.0066
FAM211A	24.45050381	71.33433278	1.544732412	1E-04	0.0054
ZNF503-AS2	13.74156505	40.02790935	1.542459941	0.001	0.0392
GFRA1	24.45050381	71.03079517	1.538580443	1E-04	0.005
MDGA1	37.4605598	108.7280979	1.537280451	5E-04	0.0172
GREM2	131.0142518	378.1097022	1.529081112	8E-11	2E-08
BASP1	416.3367788	1197.5204	1.524227317	3E-14	1E-11
PRKD1	95.90810421	275.0411305	1.519922747	1E-05	0.0007
KHDRBS2	20.63307342	58.56688843	1.505126513	6E-04	0.0195
ADRB2	30.16776688	85.21005373	1.498015753	3E-05	0.0017
GPD1	17.57676045	49.48074839	1.493198128	0.001	0.0426
ACTN3	2098.489449	5865.522107	1.482908323	6E-17	3E-14
PLAT	29.41257114	82.07675302	1.480540754	7E-05	0.0036
CRABP2	757.5347252	2092.755564	1.466019885	5E-14	2E-11
CD4	37.78486265	104.2990813	1.464846167	5E-06	0.0003
DUSP6	213.1748692	585.3226342	1.457194697	2E-05	0.0013
FBXO40	732.5066757	1967.707085	1.425601663	8E-13	2E-10
RP11-111H3.1	33.63720774	89.99554486	1.419795633	2E-04	0.0072
CTD-3083F21.5	27.48313011	73.17805229	1.412864692	1E-04	0.0048
GATA2	22.54474945	59.85442474	1.408666426	0.001	0.0339
FOSB	22.15530824	58.81135443	1.408442317	9E-04	0.0282
SERPING1	307.0765507	797.8749653	1.377564332	2E-11	6E-09
CXCL12	109.9858155	283.6097099	1.366589451	2E-04	0.0068
RP11-31F19.1	40.89447064	105.0406589	1.360970177	9E-05	0.0042
CACNA1G	106.4808446	273.0079268	1.358348921	1E-08	1E-06
HOXB3	105.8381606	270.4960155	1.35374745	1E-06	9E-05
C1orf54	55.00179023	138.6165724	1.333549268	1E-05	0.0007
LRRTM4	32.84648199	82.38847022	1.326703595	2E-04	0.0084
PDE3A	26.75162104	66.36549216	1.310806967	0.001	0.0389
MAPK10	78.76815832	193.6305242	1.29762195	2E-05	0.0011
CAPN3	249.5914605	613.2574256	1.296924223	6E-04	0.0198
RIMBP2	133.6574369	327.280301	1.291986656	2E-08	3E-06
ZNF385B	51.57380105	125.6657786	1.284881544	4E-05	0.0023
TNFAIP3	70.30112012	171.1575953	1.283705733	8E-06	0.0005
SORCS1	931.5860687	2254.104306	1.274793306	5E-05	0.0025
KLHL14	922.1328897	2228.34926	1.272928792	4E-10	6E-08
NFYB	969.6833909	2334.577716	1.267575937	2E-06	0.0002
ZNF503	148.9034717	357.4296007	1.263281726	5E-05	0.0025
BIN1	275.4339224	655.8399587	1.251637552	4E-09	5E-07
THBS4	366.3385076	869.4396803	1.246908583	2E-07	2E-05
CCDC80	597.5861685	1418.187372	1.246829494	4E-10	8E-08
TBKBP1	1803.676458	4279.913426	1.246641042	7E-12	2E-09

CPXM1	547.6694117	1298.834997	1.245840953	1E-10	2E-08
MAF	184.9543084	436.1631198	1.237698879	5E-06	0.0003
BOC	58.45346608	137.5205652	1.2342869	1E-04	0.0049
PPAP2B	390.9015231	919.5906653	1.234186615	3E-09	4E-07
ADCY8	139.1096138	326.2208715	1.229626961	9E-07	8E-05
MYH6	82016.35376	191890.2152	1.226297636	2E-11	5E-09
MTMR9LP	37.08296193	86.56279633	1.222990624	0.001	0.0361
MN1	192.8128037	447.2947329	1.214024914	1E-08	2E-06
ST6GALNAC3	61.89922027	143.5283864	1.213342953	7E-05	0.0033
RNF157	212.3249268	490.8714891	1.209071622	2E-08	2E-06
C7	750.9807519	1733.562225	1.206891786	8E-04	0.0266
SEMA7A	146.6852578	336.1117505	1.196217096	4E-07	4E-05
PBX3	676.3884679	1531.700961	1.179210695	5E-10	9E-08
CA11	192.4529708	430.1023618	1.160174112	8E-08	9E-06
GRIK5	40.11558823	89.44958614	1.156911855	9E-04	0.0279
HTR1B	52.718438	117.3191713	1.154059255	2E-04	0.0097
TMEM41A	535.7757733	1187.026536	1.147650934	3E-08	3E-06
ADAMTS15	209.6935851	463.6416976	1.144727594	4E-04	0.0139
ADCY1	568.3617018	1253.771227	1.141392877	3E-04	0.0108
FOXH1	221.1044245	487.7361435	1.141372992	9E-08	1E-05
MAT1A	86.4030191	189.1403911	1.130303856	1E-04	0.0058
COLEC12	199.6450953	435.3279378	1.12466498	4E-07	3E-05
DGKI	715.0365053	1556.529036	1.122243685	2E-08	3E-06
SUSD4	133.6159852	290.1859836	1.118885222	1E-06	8E-05
RBM38	2628.552126	5700.958729	1.116936213	2E-09	3E-07
PEG10	5784.383012	12499.86816	1.11167789	3E-09	5E-07
CPT1C	84.06045018	181.2256891	1.108288387	7E-05	0.0035
ZFP36	137.4393372	296.2692356	1.108113841	1E-06	9E-05
GLUL	1838.493832	3956.450976	1.105682544	1E-09	2E-07
PDE4A	72.20095281	155.067366	1.102805322	1E-04	0.006
ABHD6	236.5103445	505.900244	1.09694965	1E-05	0.0008
VWC2	55.80435932	119.3441954	1.096678667	9E-04	0.0278
HOXB-AS1	188.3290026	401.8763021	1.093496316	2E-05	0.001
SMAD9	679.782316	1448.860367	1.091773826	4E-06	0.0003
ELAVL2	166.6105089	354.540766	1.089472121	5E-06	0.0003
COLQ	255.1784468	538.6795981	1.077920946	6E-05	0.003
RP11-54O7.3	84.43212638	177.6666178	1.073308682	1E-04	0.0046
ADAMTS3	232.2560995	488.6101786	1.072967433	8E-07	7E-05
LMO3	284.7805114	598.5769657	1.071686342	3E-04	0.0122
SMPDL3B	64.92000323	135.4221552	1.060728808	3E-04	0.0103
TGFB2	2970.586416	6171.418228	1.054854309	5E-05	0.0027
CASQ1	194.040344	399.6352102	1.042327057	2E-04	0.0069
NCALD	136.7611232	281.6030899	1.042004988	2E-05	0.001
ASB11	81.04558888	165.8097863	1.032723588	1E-03	0.0311
DDN	256.5940916	524.0725241	1.030278523	3E-05	0.0015
PIPOX	60.36514211	123.0825264	1.027838352	0.001	0.0332
DYNC1I1	135.9822408	277.2392796	1.027713426	2E-05	0.0012

Development • Supplementary information

ID1	300.1968857	611.2607997	1.025879039	7E-07	6E-05
FIBIN	83.31117611	169.3138758	1.023118261	4E-04	0.0154
CPS1	1026.598246	2082.070631	1.02014731	4E-04	0.0164
NR4A1	268.400294	542.8009873	1.016037093	2E-06	0.0001
MEIS2	2710.111511	5470.051459	1.013202191	3E-08	4E-06
GRIA1	64.93776824	130.9483813	1.011868518	6E-04	0.0209
ART5	153.4997845	309.3443073	1.010976855	8E-06	0.0005
TSPAN7	361.0817457	726.7953231	1.009223648	2E-06	0.0002

Development • Supplementary information

Supplemental Table 3. Quantitative PCR Primers for human genes

Gene Name	Forward Primer	Reverse Primer
HPRT	TGACACTGGCAAAACAATGCA	GGTCCTTTTCACCAGCAAGCT
TMEM88	GCTGCCTTCAATCTTCTCCTG	ATAAAGGGCTCGGCTGTAGG
TNNT2	TTCACCAAAGATCTGCTCCTCGCT	TTATTACTGGTGTGGAGTGGGTGTGG
T	CAAATCCTCATCCTCAGTTTG	GTCAGAATAGGTTGGAGAATTG
ISL1	ATTTCCCTATGTGTTGGTTGC	CGTTCTTGCTGAAGCCGATG
MYH6	CAAGTTGGAAGACGAGTGCT	ATGGGCCTCTTGTAGAGCTT
MYL4	TCAAAGAGGCCTTTTCATTG	CGTCTCAAAGTCCAGCATCT
MYL7	TCCAACGTCTTTTCCATGTT	TCTGTCCCATTGAGCTTCTC
SCL	AAGGGCACAGCATCTGTAGTCA	AAGTCTTCAGCAGAGGGTCACGTA
NODAL	TGGAGGTGGGATGAAGTCACCTAT	AACCCAGCCTGAGGCAATGAGATT
DKK1	AACAGCTATCCAAATGCAG	TCACAGGGGAGTTCCATAAA
WNT5a	TAGCAGCATCAGTCCACAAA	CAAAACACGGCATCTCTCTT
αSMA	TCCCTTGAGAAGAGTTACGA	CCCCTGATAGGACATTGTTA
HAND2	AGATCAAGAAGACCGACGTG	TCCTTCTCCTCCTCCTCACT
HAND1	TCAAAGACGCACTCTTCCAC	GTGCAGCGACAAAAAGAAAA
GATA1	CTTTCAGGTGTACCCATTGC	AAAGTCTCCAGGAAGCTGGT
NKX2.5	CCAAGGACCCTAGAGCCGAA	ATAGGCGGGGTAGGCGTTAT
TBX5	GAACCACAAGATCACGCAATTA	ACACCATTCTCACACTGGTAT
ATP2A2	ATGACAACCCACTGAGAAGAGAA	CGAAGGTCAGATTGGTCTCATATTT
RYR2	AGAACTTACACACACGCGACCTG	CATCTCTAACCGGACCATACTGC
CDX1	GGTGGCAGCGGTAAGACTC	TGTAACGGCTGTAATGAAACTCC
GATA4	ACACCCCAATCTCGATATGTTTG	GTTGCACAGATAGTGACCCGT
CD34	AAATCCTCTTCCTCTGAGGCTGGA	AAGAGGCAGCTGGTGATAAGGGTT
CD31	ATCATTTCTAGCGCATGGCCTGGT	ATTTGTGGAGGGCGAGGTCATAGA
GSC	GAGGAGAAAGTGGAGGTCTGGTT	CTCTGATGAGGACCGCTTCTG
CD144	TGGAGAAGTGGCATCAGTCAACAG	TCTACAATCCCTTGCAGTGTGAG
NFATC1	GCATTTTCCTTGATCCCTGT	AGCAGCTTTAGGGTGCAAAT
MESP1	TCGAAGTGGTTCCTTGGCAGAC	CCTCCTGCTTGCCTACAAAGTGTC
KDR	ATGCACGGCATCTGGGAATC	GTCACTGTCCTGCAAGTTGCTGTC
WNT8a	GCAGAGGCGGAACTGATCTT	CGACCCTCTGTGCCATAGATG
WNT3a	AACTACGTGGAGATCATGCCC	GACTCCCTGGTAGCTTTGTC
RUNX1	ATGTGGTCCTATTTAAGCCAGCCC	TCATCTGGCTGAAGACACCAGCTT
EFNB2	CTTTTAGGATGGGGGAGAGG	TGACCAGGGACGATCATACA
CXCR4	GGTGGTCTATGTTGGCGTCT	TGGAGTGTGACAGCTTGGAG
SOX17	TTTTGTCTGCCACTTGAACA	CCCAGGACAACATTTCTTTG

Development • Supplementary information

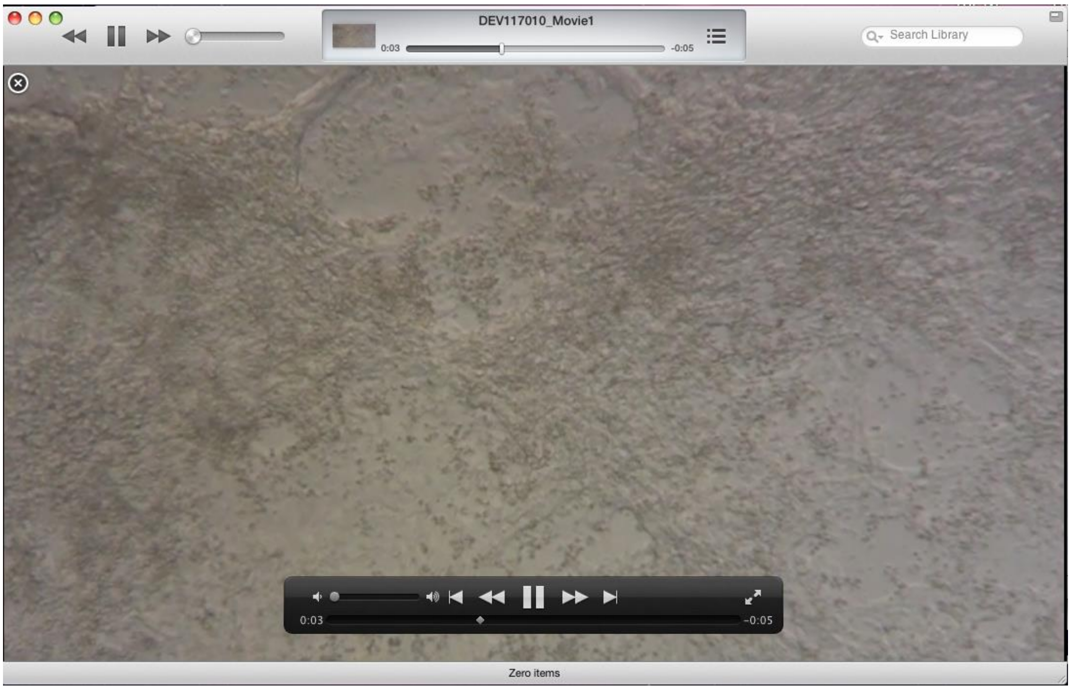

Movie 1. Day 14 beating cardiomyocytes generated from A^{100}/B^{5}-EC^{XAV}.

Development • Supplementary information
